# Convergent generation of atypical prions in knockin mouse models of genetic prion disease

**DOI:** 10.1172/JCI176344

**Published:** 2024-08-01

**Authors:** Surabhi Mehra, Matthew E.C. Bourkas, Lech Kaczmarczyk, Erica Stuart, Hamza Arshad, Jennifer K. Griffin, Kathy L. Frost, Daniel J. Walsh, Surachai Supattapone, Stephanie A. Booth, Walker S. Jackson, Joel C. Watts

**Affiliations:** 1 Tanz Centre for Research in Neurodegenerative Diseases and; 2 Department of Biochemistry, University of Toronto, Toronto, Ontario, Canada.; 3 Wallenberg Center for Molecular Medicine, Department of Biomedical and Clinical Sciences, Linköping University, Linköping, Sweden.; 4 German Center for Neurodegenerative Diseases (DZNE), Bonn, Germany.; 5 One Health Division, National Microbiology Laboratory, Public Health Agency of Canada, Winnipeg, Manitoba, Canada.; 6 Department of Biochemistry and Cell Biology and; 7 Department of Medicine, Geisel School of Medicine at Dartmouth, Hanover, New Hampshire, USA.; 8 Department of Medical Microbiology and Infectious Diseases, Faculty of Health Sciences, University of Manitoba, Winnipeg, Manitoba, Canada.

**Keywords:** Neuroscience, Neurodegeneration, Prions

## Abstract

Most cases of human prion disease arise due to spontaneous misfolding of WT or mutant prion protein, yet recapitulating this event in animal models has proven challenging. It remains unclear whether spontaneous prion generation can occur within the mouse lifespan in the absence of protein overexpression and how disease-causing mutations affect prion strain properties. To address these issues, we generated knockin mice that express the misfolding-prone bank vole prion protein (BVPrP). While mice expressing WT BVPrP (I109 variant) remained free from neurological disease, a subset of mice expressing BVPrP with mutations (D178N or E200K) causing genetic prion disease developed progressive neurological illness. Brains from spontaneously ill knockin mice contained prion disease–specific neuropathological changes as well as atypical protease-resistant BVPrP. Moreover, brain extracts from spontaneously ill D178N- or E200K-mutant BVPrP–knockin mice exhibited prion seeding activity and transmitted disease to mice expressing WT BVPrP. Surprisingly, the properties of the D178N- and E200K-mutant prions appeared identical before and after transmission, suggesting that both mutations guide the formation of a similar atypical prion strain. These findings imply that knockin mice expressing mutant BVPrP spontaneously develop a bona fide prion disease and that mutations causing prion diseases may share a uniform initial mechanism of action.

## Abstract 2

Knock-in mouse models that express the misfolding-prone bank vole prion protein provide insight into the etiology of genetic prion diseases.

## Graphical abstract

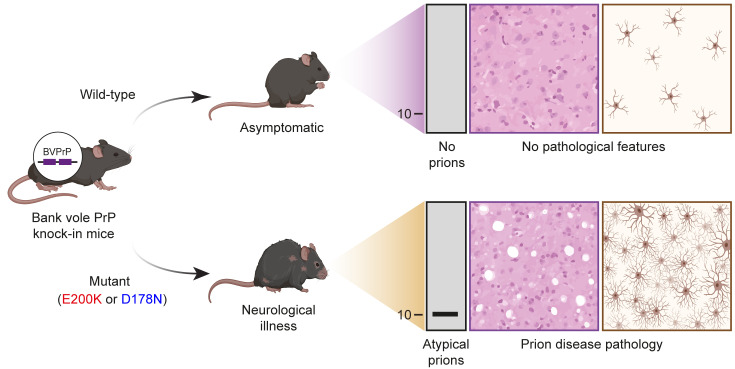

## Introduction

Human prion diseases such as Creutzfeldt-Jakob disease (CJD) are caused by misfolding of the cellular prion protein (PrP^C^) into PrP^Sc^, a pathological conformation that aggregates and deposits in the brain (1). In addition to PrP^Sc^ deposition, the neuropathological hallmarks of prion disease include spongiform degeneration of the brain parenchyma as well as prominent astrocytic gliosis (2). PrP^C^ is expressed at its highest levels in neurons and astrocytes, can be modified by the addition of up to two N-linked glycans, and is attached to the outer leaflet of the plasma membrane by a glycosylphosphatidylinositol (GPI) anchor. While PrP^C^ is predominantly α-helical, PrP^Sc^ aggregates adopt a parallel in-register β-sheet structure and are partially resistant to degradation by proteases such as proteinase K (PK) (3–6). Structural variation within PrP^Sc^ aggregates encodes distinct prion strains, which produce unique disease phenotypes (7–10). Aggregates of PrP^Sc^ can act as seeds to template the conformational conversion of PrP^C^ into additional PrP^Sc^, allowing prions to spread from cell to cell within the brain and from the periphery into the central nervous system.

The ability of PrP^Sc^ to self-propagate underlies the infectious nature of the prion diseases. However, most cases of prion disease in humans do not manifest due to an infectious etiology. Instead, spontaneous misfolding of PrP^C^ into PrP^Sc^ within the brain is thought to be the initiating event in sporadic prion diseases, such as sporadic CJD (sCJD). Similarly, in genetic prion diseases such as familial CJD (fCJD), fatal familial insomnia (FFI), and Gerstmann-Sträussler-Scheinker disease (GSS), mutations within the *PRNP* gene encoding PrP are believed to promote the spontaneous formation of PrP^Sc^. Different disease-causing mutations in human PrP can lead to the formation of distinct prion strains that exhibit differential susceptibility to cleavage by PK (11). The biochemical hallmark of sCJD, fCJD, and FFI is “classical” protease-resistant PrP (PrP^res^), also called PrP27-30, which is a C-terminal fragment characterized by multiple PK-resistant glycoforms with molecular weights between 19 and 30 kDa (12, 13). In contrast, “atypical” N- and C-terminally truncated PrP^res^ fragments with molecular weights between about 6 and 11 kDa are found in most cases of GSS (14–16).

Although about 99% of human prion disease cases arise due to the spontaneous generation of PrP^Sc^ within the brain, little is known about the molecular mechanisms that govern this process. Inoculation of WT or transgenic mice with prions induces a neurodegenerative disorder that recapitulates all the biochemical and pathological hallmarks of human prion diseases (17). However, it remains unknown whether the earliest events that occur in sporadic and genetic prion diseases are the same as those in infectious prion diseases, which require a preexisting source of PrP^Sc^. Thus, there has been considerable interest in developing mouse models that exhibit spontaneous prion formation within the brain. While some success has been achieved, no existing model fully recapitulates the key features present in the human prion diseases (18, 19). Certain lines of transgenic mice overexpressing mouse PrP (MoPrP) containing prion disease–causing pathogenic mutations develop spontaneous neurological illness but fail to generate infectious PrP^Sc^ that is highly resistant to PK digestion (20–29). Similarly, knockin mouse models of fCJD, FFI, and GSS do not exhibit overt signs of neurological illness nor highly PK-resistant PrP despite the presence of prion disease–specific neuropathological changes within the brains of certain lines (30–32). Interestingly, transgenic mice overexpressing mutant human PrP also fail to develop neurological illness, suggesting that sequence elements within human PrP prevent spontaneous misfolding within the lifespan of a mouse (33, 34).

In recent years, bank voles (*Myodes glareolus*) have been increasingly used in prion disease research, since they are susceptible to prion strains from several different species, including humans (35–40). Bank vole PrP (BVPrP) facilitates prion replication and formation in various in vitro, cellular, and animal paradigms, indicating that BVPrP is a highly permissive substrate for conversion into PrP^Sc^ (41–51). Moreover, transgenic mice overexpressing WT BVPrP containing isoleucine at polymorphic codon 109 (I109) develop a spontaneous and transmissible neurological illness characterized by prion disease–specific neuropathological changes as well as the presence of a highly PK-resistant PrP fragment in the brain (52–54). Addition of the D178N or E200K mutations, which are common causes of genetic prion disease, to the sequence of BVPrP(I109) hastens the onset of neurological disease in transgenic mice (53). Collectively, these findings suggest that BVPrP(I109) is intrinsically prone to adopting misfolded, infectious conformations, potentially making it an ideal substrate for studying spontaneous prion formation under conditions of physiological protein expression.

In this study, we sought to generate improved mouse models of spontaneous prion formation by taking advantage of the unique misfolding propensity of BVPrP as well as the more translational features of knockin mice, including physiological levels of PrP expression with the correct spatiotemporal pattern within the brain. Whereas knockin mice expressing WT BVPrP(I109) remained healthy for their entire lifespan, knockin mice expressing either D178N- or E200K-mutant BVPrP(I109) developed progressive signs of neurological illness, which were accompanied by the emergence of atypical PrP^res^ within the brain. Furthermore, brain extracts from spontaneously ill mutant BVPrP(I109)–expressing knockin mice transmitted disease to knockin mice expressing WT BVPrP(I109), confirming the generation of authentic prion infectivity. Surprisingly, stark differences in disease phenotype were not observed between the D178N- and E200K-mutant lines, implying that both mutations may initially promote the formation of a similar, perhaps identical, atypical prion strain.

## Results

### Generation of BVPrP-knockin mice.

**Figure 1:**
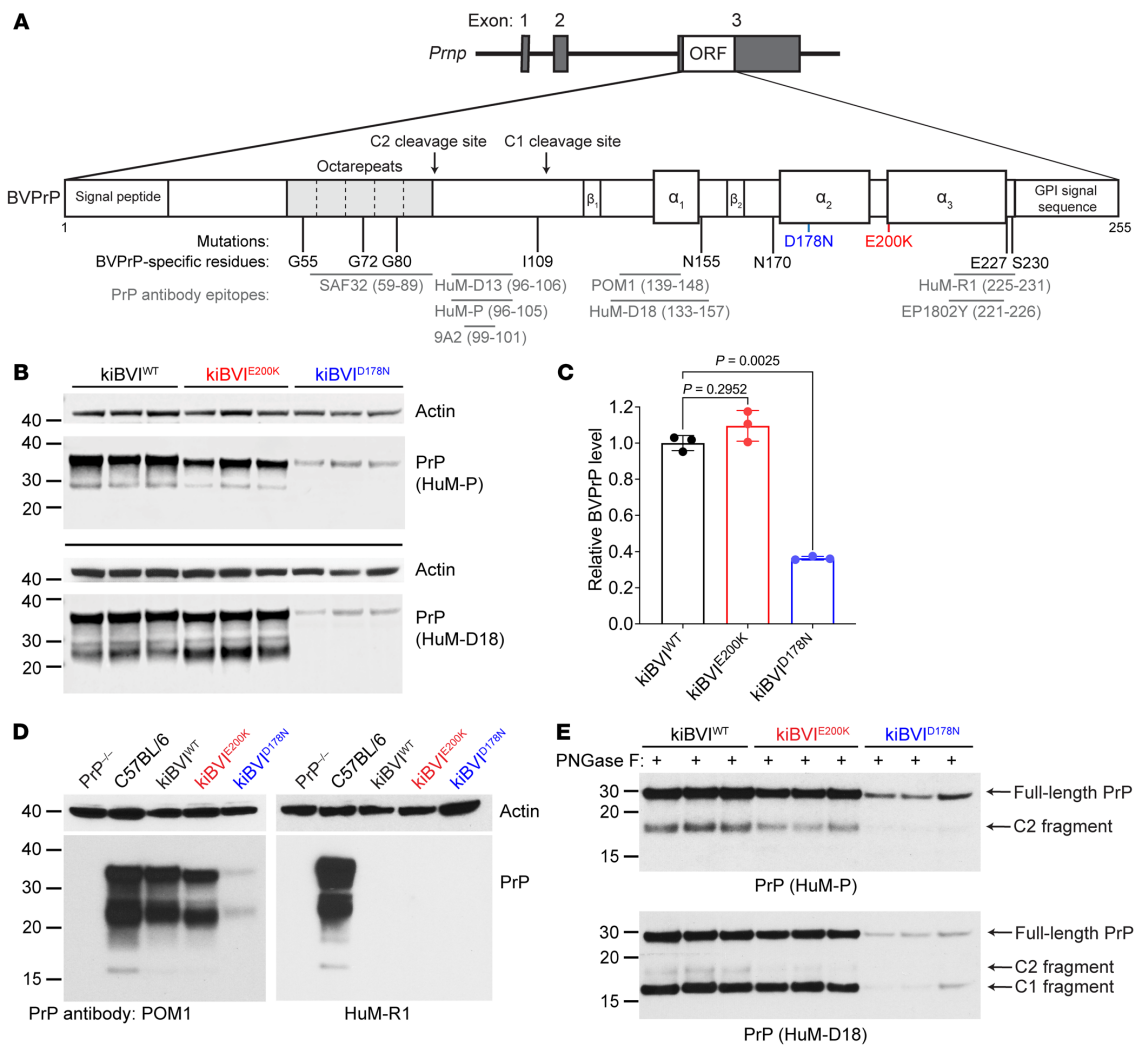
Generation and characterization of knockin mice expressing WT or mutant bank vole PrP. (**A**) Schematic of the gene-targeted alleles in knockin mice expressing either WT (kiBVI^WT^), E200K-mutant (kiBVI^E200K^), or D178N-mutant (kiBVI^D178N^) BVPrP(I109). The 8 amino acid residue differences between the mature forms of bank vole and mouse PrP are shown, as are the approximate epitopes for the anti-PrP antibodies used in this study. (**B**) Immunoblots for PrP in brain extracts from 3 mice each for the indicated knockin lines. BVPrP was detected using the antibodies HuM-P and HuM-D18, and both blots were reprobed with an anti-actin antibody. (**C**) ELISA-based quantification of relative BVPrP levels (mean ± SD) in brain extracts from knockin mice (*n* = 3 per line). Statistical significance was assessed using Welch’s ANOVA followed by Dunnett’s T3 multiple-comparison test. (**D**) Immunoblots for PrP in brain extracts from the indicated mouse lines probed with antibodies that recognize both mouse and bank vole PrP (POM1) or only mouse PrP (HuM-R1). Both blots were reprobed with an antibody against actin. (**E**) Immunoblots for PrP in PNGase F–treated brain extracts from 3 mice each for the indicated knockin lines. BVPrP was detected using the antibodies HuM-P and HuM-D18. Full-length BVPrP as well as the C1 and C2 endoproteolytic products are indicated. In all panels, the molecular weight markers indicate kilodaltons.

To generate mice expressing physiological levels of WT or mutant BVPrP, we replaced the open reading frame encoding MoPrP, which is located entirely within the third exon of the *Prnp* gene, with that of BVPrP using a CRISPR/Cas9–based gene editing approach (Figure 1A) (55). To promote spontaneous prion formation in the brain, we used the I109 polymorphic variant of BVPrP, since transgenic mice overexpressing WT BVPrP(I109) develop spontaneous disease much more rapidly than mice overexpressing BVPrP with methionine at codon 109 (M109) (52, 56). We have previously found that adding the D178N and E200K mutations to BVPrP(I109) hastens the onset of spontaneous neurological illness in transgenic mice (53). Thus, we generated knockin mice expressing either WT, D178N-mutant, or E200K-mutant BVPrP(I109), which will be referred to as kiBVI^WT^, kiBVI^D178N^, and kiBVI^E200K^ mice, respectively. All mice were homozygous for the knockin alleles and thus expressed only WT or mutant BVPrP(I109). The E200K mutation causes fCJD when paired with either methionine (M) or valine (V) at polymorphic codon 129 in human PrP (57, 58). In contrast, the D178N mutation typically causes FFI if it occurs in *cis* to M129 or fCJD if it occurs in *cis* to V129 (59). However, the D178N-M129 haplotype can occasionally cause a CJD-like phenotype (60, 61). Since BVPrP contains methionine at codon 129, the kiBVI^D178N^ mice are potentially more reflective of FFI than fCJD.

BVPrP(I109) expression levels in the brain differed among the 3 lines, with relative levels dependent on the anti-PrP antibody used for detection by immunoblotting (Figure 1B and Supplemental Figure 1A; supplemental material available online with this article; https://doi.org/10.1172/JCI176344DS1). PrP levels in kiBVI^E200K^ mice were similar to or slightly lower than levels in kiBVI^WT^ mice, whereas levels in kiBVI^D178N^ mice were considerably lower. Quantification of relative BVPrP(I109) expression levels in the brain by ELISA revealed that PrP levels were approximately 50%–60% lower in kiBVI^D178N^ mice than in kiBVI^WT^ mice but similar between kiBVI^E200K^ and kiBVI^WT^ mice (Figure 1C). This is consistent with results from MoPrP-based knockin mice and our previous finding that D178N-mutant BVPrP(I109) has a shorter half-life than either WT or E200K-mutant BVPrP(I109), resulting in lower steady-state levels (31, 53). Brain BVPrP(I109) levels in kiBVI^WT^ mice were comparable to MoPrP levels in the brains of WT C57BL/6 mice, and PrP was not detected in any of the knockin lines when an antibody that recognizes MoPrP but not BVPrP was used (Figure 1D). Following the removal of N-linked glycans, endoproteolytic processing of BVPrP(I109) to produce the C1 and C2 fragments was observed in all 3 lines (Figure 1E and Supplemental Figure 1B). This indicates that PrP^C^ synthesis and processing occur similarly in WT mice and the BVPrP(I109)-expressing knockin lines.

### Mutant BVPrP–knockin mice develop spontaneous disease.

**Figure 2:**
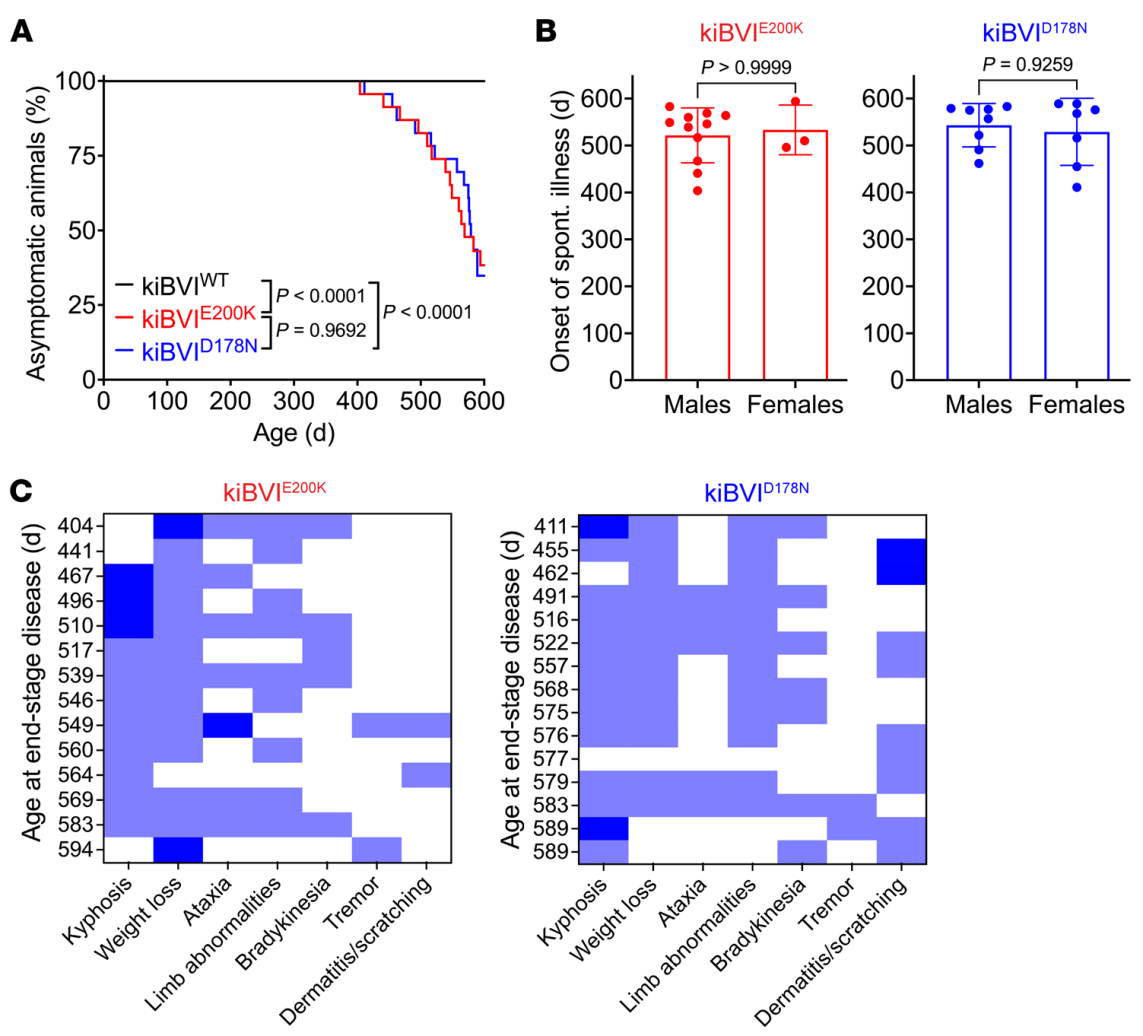
Knockin mice expressing mutant bank vole PrP develop spontaneous neurological illness. (**A**) Kaplan-Meier curves for the development of signs of neurological illness in kiBVI^WT^ (black, *n* = 22), kiBVI^E200K^ (red, *n* = 23), and kiBVI^D178N^ (blue, *n* = 23) mice. Statistical significance was assessed using the log-rank test. (**B**) Analysis of sex-specific effects on the age of onset of spontaneous neurological illness (mean ± SD) in kiBVI^E200K^ (left graph; *n* = 11 for males, *n* = 3 for females) and kiBVI^D178N^ (right graph; *n* = 8 for males, *n* = 7 for females) mice. Statistical significance was assessed using unpaired, 2-tailed Mann-Whitney tests. (**C**) Heatmaps for commonly observed signs of neurological illness in kiBVI^E200K^ (left, *n* = 14) and kiBVI^D178N^ (right, *n* = 15) mice that developed spontaneous disease at the indicated ages. Each row represents a single mouse, and symptoms were designated as absent (white; score = 0), moderate (light blue; score = 1), or severe (dark blue; score = 2).

Groups of kiBVI^WT^, kiBVI^D178N^, and kiBVI^E200K^ mice were monitored longitudinally for the development of signs of spontaneous neurological illness consistent with prion disease. Mice were analyzed up to 20 months of age since nonspecific causes of morbidity or mortality in aged mice appear more frequently after this point. Whereas all kiBVI^WT^ mice remained free of neurological illness for the duration of the experiment, a subset of kiBVI^D178N^ and kiBVI^E200K^ mice developed progressive signs of neurological disease beginning around 400 days of age (Figure 2A and Supplemental Table 1). Approximately 60% of kiBVI^D178N^ and kiBVI^E200K^ mice developed spontaneous disease by 20 months of age, and there was no difference in the kinetics of disease onset between the 2 mutant BVPrP-expressing lines, nor was the age of disease onset sex-dependent (Figure 2B). Even accounting for mice that developed intercurrent illness (Supplemental Table 2), all-cause mortality was significantly increased in aged kiBVI^D178N^ and kiBVI^E200K^ mice compared with kiBVI^WT^ mice (Supplemental Figure 2A). In spontaneously sick kiBVI^E200K^ and kiBVI^D178N^ mice, the most common signs of illness were prominent kyphosis and weight loss with the variable presence of limb abnormalities and ataxia (Figure 2C). Some mice also exhibited a severe dermatitis phenotype characterized by neurotic scratching/overgrooming of the head region, and this was more common in the kiBVI^D178N^ line. Progressive weight loss tended to begin approximately 3–4 weeks before terminal disease (Supplemental Figure 2B).

### Progressive accumulation of misfolded PrP species in mutant BVPrP–knockin mice.

**Figure 3:**
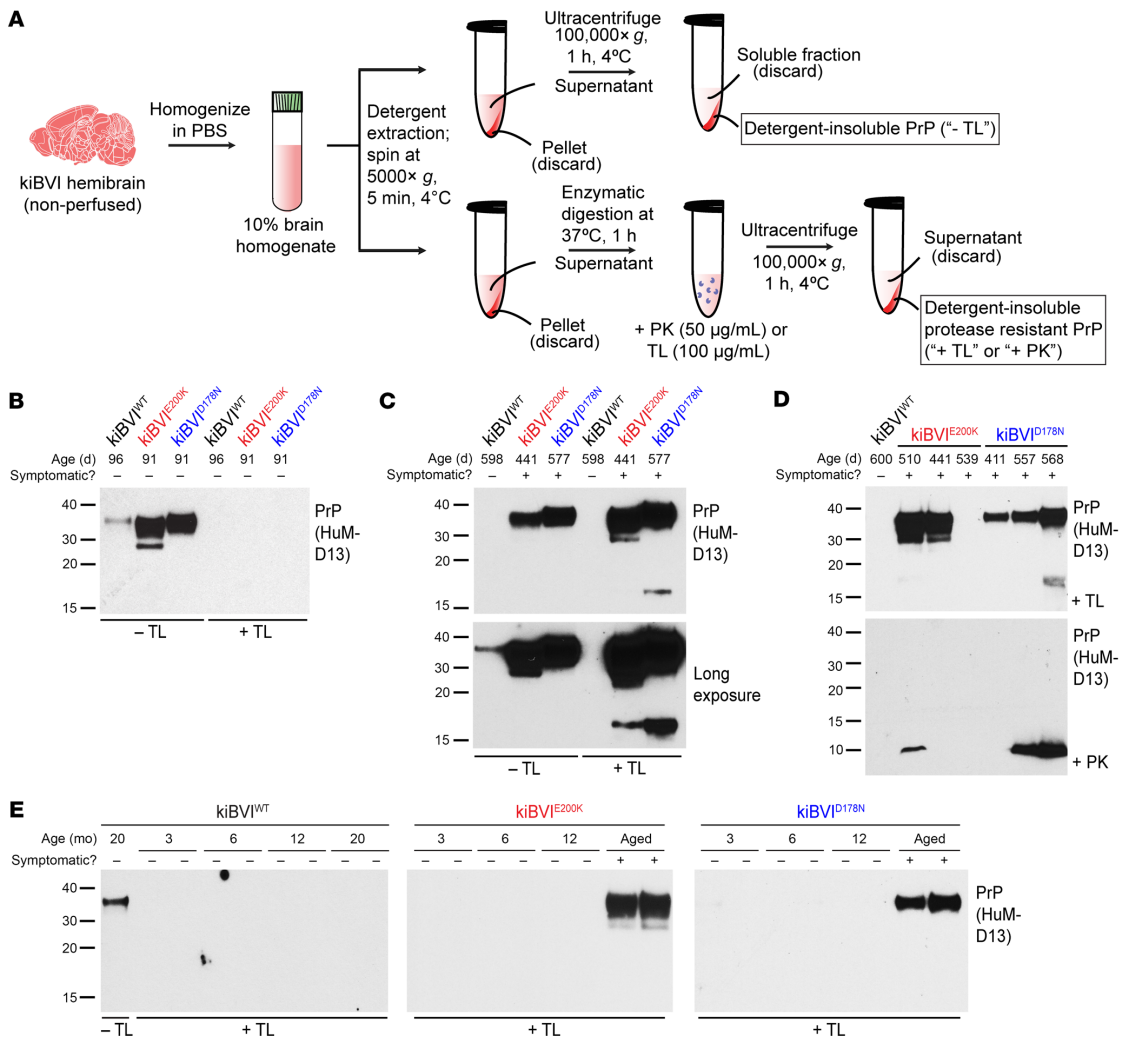
Protease-resistant PrP in brains from spontaneously ill knockin mice expressing mutant bank vole PrP. (**A**) Schematic of protocols used for the analysis of detergent-insoluble and protease-resistant PrP species in the brains of knockin mice. (**B**) Immunoblots for detergent-insoluble PrP species, with (+) or without (–) digestion with thermolysin (TL), in brain homogenates from 3-month-old kiBVI^WT^, kiBVI^E200K^, and kiBVI^D178N^ mice. (**C**) Immunoblots for detergent-insoluble PrP species, with or without TL digestion, in brain homogenates from asymptomatic 20-month-old kiBVI^WT^ mice and spontaneously ill kiBVI^E200K^ and kiBVI^D178N^ mice. The bottom panel displays a longer exposure of the blot shown in the top panel. In the immunoblots in **C** and **D**, 10 times more material was loaded for the TL-digested samples than for the undigested samples. (**D**) Immunoblots for detergent-insoluble PrP species in brain extracts from aged asymptomatic kiBVI^WT^ mice as well as spontaneously ill kiBVI^E200K^ and kiBVI^D178N^ mice following digestion with either TL (top panel) or proteinase K (PK; bottom panel). (**E**) Immunoblots for detergent-insoluble, TL-resistant PrP species in brain extracts from kiBVI^WT^ (left), kiBVI^E200K^ (middle), and kiBVI^D178N^ (right) mice at the indicated ages. Two independent mice per age are shown. For the kiBVI^WT^ line, a sample without TL digestion is also shown. In all panels, PrP was detected using the antibody HuM-D13, and molecular weight markers indicate kilodaltons.

The brains of spontaneously ill transgenic mice overexpressing WT or mutant BVPrP(I109) contain atypical PrP^res^ species (53). Thus, we analyzed brain extracts from the knockin mice for the presence of detergent-insoluble and protease-resistant PrP (Figure 3A). Initially, we used the protease thermolysin (TL), which has been shown to effectively discriminate between normal and aggregated forms of proteins in prion disease and other protein misfolding disorders (28, 62–64). In brains from healthy 3-month-old knockin mice, full-length PrP species were fully degraded by TL concentrations higher than 2 μg/mL, whereas complete digestion of the C1 endoproteolytic fragment in the kiBVI^WT^ and kiBVI^E200K^ lines required TL concentrations of at least 20 μg/mL (Supplemental Figure 3, A and B). To ensure complete digestion of all properly folded PrP species, we used TL at a concentration of 100 μg/mL in all subsequent protease digestion experiments.

Despite expressing similar or lower levels of BVPrP, levels of detergent-insoluble PrP species were substantially elevated in the brains of 3-month-old kiBVI^E200K^ and kiBVI^D178N^ mice compared with kiBVI^WT^ mice (Figure 3B). At this age, all detergent-insoluble PrP species were sensitive to TL digestion. Compared with asymptomatic 20-month-old kiBVI^WT^ mice, increased amounts of detergent-insoluble PrP species were also present in brain extracts from spontaneously ill kiBVI^E200K^ and kiBVI^D178N^ mice, and these species were resistant to TL digestion (Figure 3C). TL-resistant PrP was not detected in any of the brains from asymptomatic 20-month-old kiBVI^WT^ mice (Figure 3, C and D, and Supplemental Table 1), even when lower concentrations of TL were used (Supplemental Figure 3C). In contrast, 100% of the brains from kiBVI^D178N^ mice that developed spontaneous neurological illness displayed TL-resistant PrP (Figure 3D and Supplemental Table 1). Moreover, brains from asymptomatic kiBVI^D178N^ mice collected at 20 months of age also contained TL-resistant PrP (Supplemental Figure 4). The presence of TL-resistant PrP was more variable in the kiBVI^E200K^ line, with only 64% of brains examined exhibiting clear evidence of TL-resistant species (Supplemental Table 1). The brains from some kiBVI^E200K^ mice that developed spontaneous neurological illness did not possess TL-resistant PrP (Figure 3D), possibly because of misdiagnosis, and TL-resistant PrP was variably present in brains from asymptomatic kiBVI^E200K^ mice collected at 20 months of age (Supplemental Figure 4). The main TL-resistant PrP species appeared to be similar in molecular weight to undigested PrP (Figure 3, C and D), and the slight difference in appearance between the kiBVI^E200K^ and kiBVI^D178N^ lines could reflect differences in N-glycosylation efficiency, which were also observed in PrP^C^ species from young mice (Figure 1B), or mutation-specific differences in protein charge.

For all 3 knockin lines, total PrP levels remained largely unchanged between young and old mice (Supplemental Figure 5A), whereas levels of detergent-insoluble PrP were modestly increased in spontaneously ill kiBVI^E200K^ and kiBVI^D178N^ mice compared with younger mice (Supplemental Figure 5, B and C). TL-resistant PrP was absent in kiBVI^E200K^ and kiBVI^D178N^ mice up to 12 months of age, suggesting that it may arise relatively late in the disease course (Figure 3E). The brains of mice with high levels of TL-resistant PrP were also examined for the presence of PK-resistant PrP species. A subset of mice examined exhibited a PK-resistant PrP fragment with a molecular weight of approximately 10 kDa (Figure 3D). As with TL digestion, PK-resistant PrP species were more commonly found in brains from the kiBVI^D178N^ line than the kiBVI^E200K^ line (Supplemental Table 1). A less abundant approximately 17-kDa TL-resistant fragment was occasionally observed in spontaneously ill kiBVI^E200K^ and kiBVI^D178N^ mice (Figure 3C). As this TL-resistant fragment was only observed in mice that also exhibited PK-resistant PrP, we speculate that it possesses a similar C-terminus to the approximately 10-kDa PK-resistant PrP fragment but with an intact N-terminus, since there are no potential TL cleavage sites in the N-terminal domain of PrP (Supplemental Figure 3A). Collectively, these results suggest that PrP^res^ progressively accumulates with age in the brains of kiBVI^E200K^ and kiBVI^D178N^ mice, but not kiBVI^WT^ mice.

### Prion disease–specific neuropathological changes in mutant BVPrP–knockin mice.

**Figure 4:**
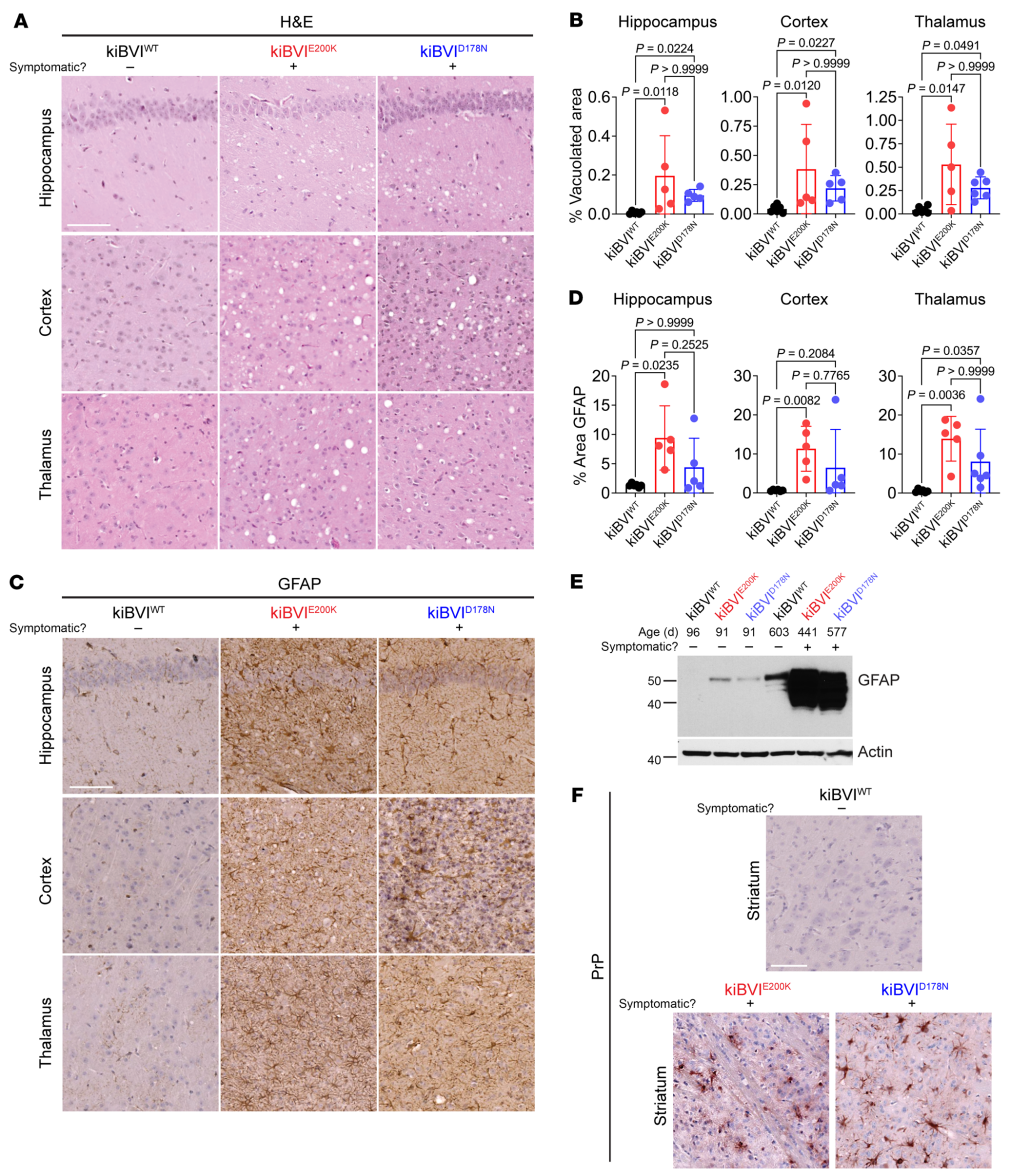
Spontaneously ill knockin mice expressing mutant bank vole PrP exhibit prion disease–specific neuropathology. (**A**) Representative H&E-stained brain sections of the hippocampus, cortex, and thalamus from spontaneously ill kiBVI^E200K^ and kiBVI^D178N^ mice as well as 20-month-old asymptomatic kiBVI^WT^ mice. Scale bar: 100 μm (applies to all sections). (**B**) Quantification of percentage area covered by vacuolation (mean ± SD) in the indicated brain regions from aged knockin mice (*n* = 5–6 samples per line). (**C**) Representative GFAP-stained brain sections of the hippocampus, cortex, and thalamus from spontaneously ill kiBVI^E200K^ and kiBVI^D178N^ mice as well as 20-month-old asymptomatic kiBVI^WT^ mice. Scale bar: 100 μm (applies to all sections). (**D**) Quantification of percentage area covered by GFAP staining (mean ± SD) in the indicated brain regions from aged knockin mice (*n* = 5–6 samples per line). (**E**) Immunoblot of GFAP levels in brain homogenates from the 3 lines of knockin mice at the indicated ages. The blot was reprobed with an antibody against actin. (**F**) Representative PrP-stained brain sections of the striatum from spontaneously ill kiBVI^E200K^ and kiBVI^D178N^ mice as well as 20-month-old asymptomatic kiBVI^WT^ mice. Scale bar: 100 μm (applies to all sections). Molecular weight markers in **E** indicate kilodaltons. In **B** and **D**, statistical significance was assessed using a Kruskal-Wallis test followed by Dunn’s multiple-comparison test.

Brains from spontaneously ill kiBVI^E200K^ and kiBVI^D178N^ mice exhibited prominent gray matter vacuolation in the hippocampus, cortex, and thalamus, whereas no vacuolation was observed in the brains of asymptomatic 20-month-old kiBVI^WT^ mice (Figure 4, A and B). Other than age-related white matter vacuolation, which was present in the brains of older mice regardless of symptomatic status or genotype (Supplemental Figure 6), vacuolation was absent in other brain regions. There was no difference in the extent or localization of vacuolation between symptomatic kiBVI^E200K^ and kiBVI^D178N^ mice (Figure 4B). The brains of symptomatic kiBVI^E200K^ and kiBVI^D178N^ mice also exhibited increased astrocytic gliosis compared with aged asymptomatic kiBVI^WT^ mice in the brain regions with vacuolation, as evidenced by GFAP immunolabeling (Figure 4, C and D). Although the increase in GFAP staining was less prominent for the kiBVI^D178N^ line, there were no statistically significant differences in the extent of GFAP immunolabeling between kiBVI^E200K^ and kiBVI^D178N^ mice (Figure 4D). Increased GFAP levels in aged symptomatic kiBVI^E200K^ and kiBVI^D178N^ mice compared with aged asymptomatic kiBVI^WT^ mice and young asymptomatic kiBVI^E200K^ and kiBVI^D178N^ mice were also observed by immunoblotting (Figure 4E and Supplemental Figure 7).

Immunohistochemical staining of brain sections from spontaneously ill kiBVI^D178N^ and kiBVI^E200K^ mice for PrP failed to detect any extracellular PrP^Sc^ deposition in brain regions exhibiting spongiform degeneration. However, intracellular PrP deposition within glial cells was selectively present in the striatum of sick kiBVI^D178N^ and kiBVI^E200K^ mice (Figure 4F), and there were no apparent differences in PrP deposition patterns between the 2 lines. We hypothesize that this intracellular staining may represent the uptake of misfolded PrP species by glial cells and subsequent failure of the endosomal/lysosomal system to effectively clear the aggregates.

### Transmission and seeding properties of spontaneously formed prions.

**Figure 5:**
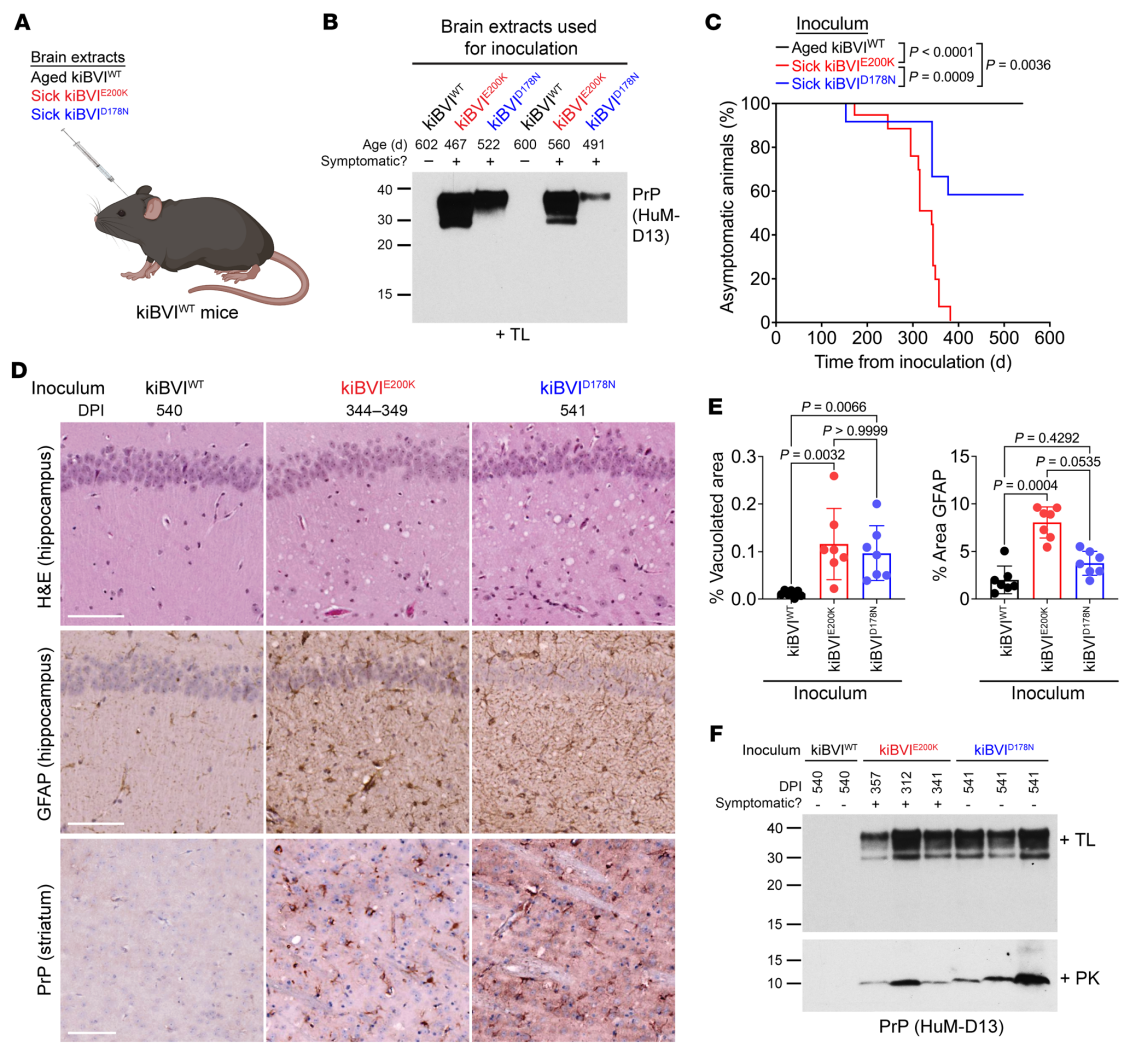
Transmission of prions from the brains of spontaneously ill knockin mice expressing mutant bank vole PrP to knockin mice expressing WT bank vole PrP. (**A**) Schematic of transmission experiments in kiBVI^WT^ mice. (**B**) Immunoblot of detergent-insoluble TL-resistant PrP levels in the 6 brain extracts used for transmission studies. (**C**) Kaplan-Meier curves for the development of neurological illness in kiBVI^WT^ mice inoculated with brain extract from aged, asymptomatic kiBVI^WT^ mice (black, *n* = 17), symptomatic kiBVI^E200K^ mice (red, *n* = 16), or symptomatic kiBVI^D178N^ mice (blue, *n* = 13). Statistical significance was assessed using the log-rank test. (**D**) Representative H&E- and GFAP-stained sections of the hippocampus and PrP-stained sections of the striatum from kiBVI^WT^ mice at the indicated days post-inoculation (DPI) with brain extract from either spontaneously ill kiBVI^E200K^ or kiBVI^D178N^ mice or 20-month-old asymptomatic kiBVI^WT^ mice. Scale bars: 100 μm. (**E**) Quantification of percentage hippocampal area covered by vacuolation or GFAP staining (mean ± SD) in the inoculated kiBVI^WT^ mice (*n* = 7 samples per line). Statistical significance was assessed using a Kruskal-Wallis test followed by Dunn’s multiple-comparison test. (**F**) Immunoblots for detergent-insoluble PrP species in brain extracts from kiBVI^WT^ mice at the indicated DPI with brain extract from kiBVI^WT^ mice, symptomatic kiBVI^E200K^ mice, or symptomatic kiBVI^D178N^ mice following digestion with either TL (top panel) or PK (bottom panel). In **B** and **F**, TL- and PK-resistant PrP species were detected using the antibody HuM-D13, and molecular weight markers indicate kilodaltons.

To test whether prion infectivity was present in the brains of spontaneously ill kiBVI^E200K^ and kiBVI^D178N^ mice, we performed transmission studies in kiBVI^WT^ mice, which do not themselves develop spontaneous disease (Figure 5A). Two inocula each from spontaneously ill kiBVI^E200K^ and kiBVI^D178N^ mice were selected based on the presence of high amounts of TL-resistant PrP in brain extracts (Figure 5B). These samples also contained PK-resistant PrP (Supplemental Figure 8). As a negative control, kiBVI^WT^ mice were inoculated with brain extracts prepared from either of 2 distinct asymptomatic 20-month-old kiBVI^WT^ mice. All the mice inoculated with kiBVI^WT^ extract remained free of neurological illness for up to 18 months after inoculation (Figure 5C and Supplemental Table 3). In contrast, 100% of kiBVI^WT^ mice inoculated with brain extracts from spontaneously ill kiBVI^E200K^ mice developed neurological illness with mean incubation periods of about 10–11 months. Disease transmission was less efficient when using inocula prepared from spontaneously ill kiBVI^D178N^ mice, possibly because of a transmission barrier introduced by the D178N mutation.

The brains of kiBVI^WT^ mice inoculated with kiBVI^E200K^ or kiBVI^D178N^ extract also contained neuropathological indicators of prion disease such as vacuolation and astrocytic gliosis as well as increased GFAP levels (Figure 5, D and E, and Supplemental Figure 9). Vacuolation in the kiBVI^WT^ mice inoculated with kiBVI^E200K^ or kiBVI^D178N^ samples was largely restricted to the hippocampus. GFAP staining in the hippocampus was less pronounced in the mice inoculated with samples from kiBVI^D178N^ mice, likely reflecting the reduced rate of symptomatic transmission. Intracellular PrP deposition within striatal glial cells was observed in kiBVI^WT^ mice inoculated with brain extract from kiBVI^E200K^ or kiBVI^D178N^ mice (Figure 5D). There were no obvious differences in the neuropathological signatures of kiBVI^WT^ mice inoculated with kiBVI^E200K^ or kiBVI^D178N^ samples. Whereas brains from kiBVI^WT^ mice inoculated with kiBVI^WT^ extract did not contain PrP^res^, TL- and PK-resistant PrP species reminiscent of those present in spontaneously ill kiBVI^E200K^ and kiBVI^D178N^ mice were detected in the brains of all kiBVI^WT^ mice inoculated with kiBVI^E200K^ extract (Figure 5F and Supplemental Table 3). Similar TL- and PK-resistant PrP species were found in the brains of some, but not all, kiBVI^WT^ mice inoculated with kiBVI^D178N^ extract. Surprisingly, of the 5 kiBVI^WT^ brains that displayed TL-resistant PrP following inoculation with kiBVI^D178N^ extract, 4 were from mice collected at 540 days after inoculation without any clinical signs of neurological illness.

**Figure 6:**
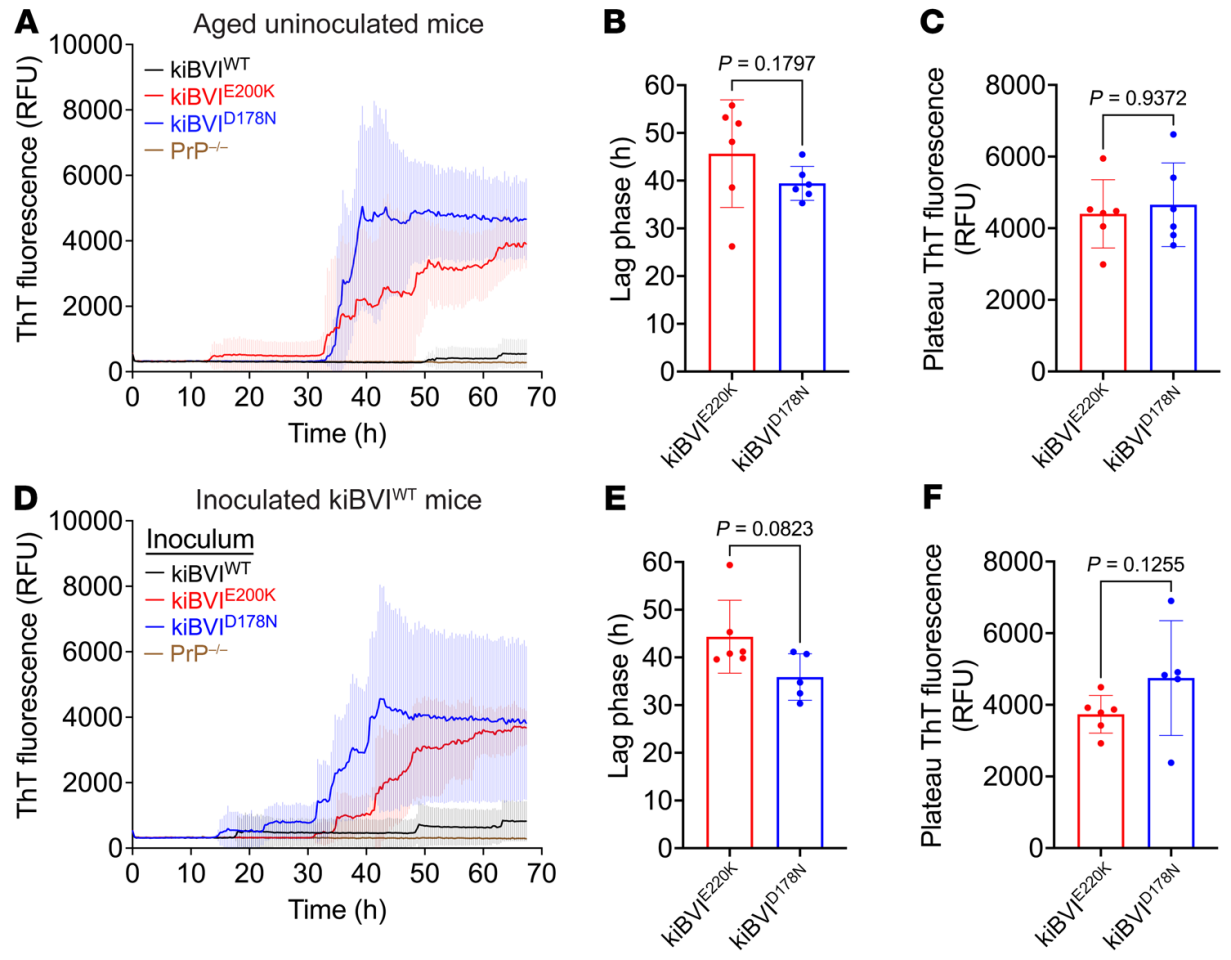
Prion seeding activity in spontaneously ill and inoculated bank vole PrP–knockin mice. (**A**) RT-QuIC assays on brain extracts (10^–4^ dilution) from spontaneously ill kiBVI^E200K^ and kiBVI^D178N^ mice as well as asymptomatic 20-month-old kiBVI^WT^ mice (*n* = 6 each). Brain extract from a PrP^–/–^ mouse was included as a negative control. Three technical replicates were performed for each brain sample. (**B** and **C**) Quantification of lag phases (**B**) and plateau ThT fluorescence values (**C**) for RT-QuIC assays on spontaneously ill kiBVI^E200K^ and kiBVI^D178N^ mice. (**D**) RT-QuIC assays on brain extracts (10^–4^ dilution) from kiBVI^WT^ mice inoculated with brain homogenate from spontaneously sick kiBVI^E200K^ or kiBVI^D178N^ mice or brain homogenate from asymptomatic aged kiBVI^WT^ mice (*n* = 6 for each group of inoculated mice). Two to three technical replicates were performed for each brain sample. (**E** and **F**) Quantification of lag phases (**E**) and plateau ThT fluorescence values (**F**) for RT-QuIC assays on inoculated kiBVI^WT^ mice. All graphs display mean ± SD. Statistical significance in **B**, **C**, **E**, and **F** was assessed using unpaired, 2-tailed Mann-Whitney tests.

We also checked whether brain extracts from spontaneously ill kiBVI^E200K^ and kiBVI^D178N^ mice exhibit prion seeding activity in the real-time quaking-induced conversion (RT-QuIC) assay. Recombinant BVPrP was used as the substrate as it can detect atypical PrP^res^ species (42). Robust seeding activity was observed in the brains of spontaneously ill kiBVI^E200K^ and kiBVI^D178N^ mice, whereas rare or no seeding activity was detected in the brains of asymptomatic 20-month-old kiBVI^WT^ mice (Figure 6A and Supplemental Table 4). The lag phases and plateau fluorescence values were indistinguishable for reactions seeded with kiBVI^E200K^ or kiBVI^D178N^ brain extract (Figure 6, B and C). Consistent results were obtained when brain extracts from inoculated kiBVI^WT^ mice were used as seeds (Figure 6, D–F, and Supplemental Table 5). Interestingly, high levels of prion seeding activity were present in brain extract from a spontaneously sick kiBVI^D178N^ mouse, approaching levels present in the brain of a C57BL/6 mouse infected with the RML strain of prions (Supplemental Figure 10). Collectively, these results demonstrate that infectious prion seeds form spontaneously in the brains of kiBVI^E200K^ and kiBVI^D178N^ mice, but not in kiBVI^WT^ mice.

### Conformational characterization of spontaneous and transmitted prions.

**Figure 7:**
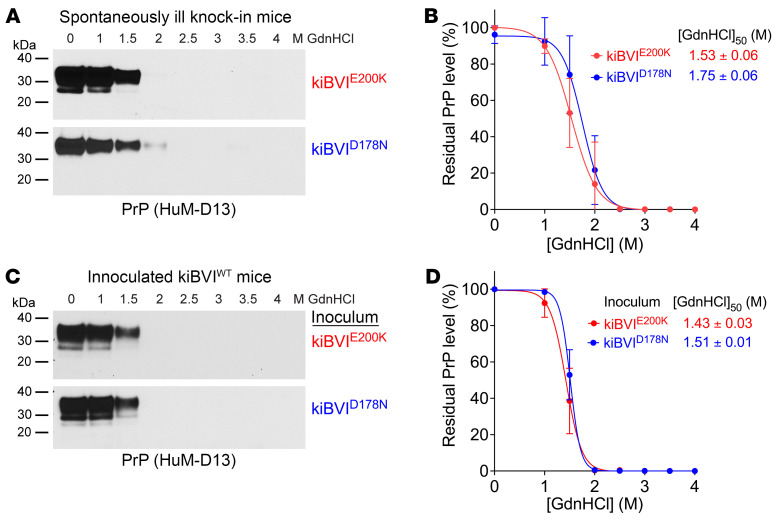
Conformational characterization of spontaneously formed and transmitted prions. (**A**) Representative immunoblots of detergent-insoluble, TL-resistant PrP species following treatment of brain extracts from spontaneously ill kiBVI^E200K^ mice (top) or kiBVI^D178N^ mice (bottom) with the indicated concentrations of guanidine hydrochloride (GdnHCl). (**B**) Quantification of residual detergent-insoluble, TL-resistant PrP levels (mean ± SD) in brain extracts from spontaneously ill kiBVI^E200K^ mice (red, *n* = 3) or kiBVI^D178N^ mice (blue, *n* = 3) treated with the indicated concentrations of GdnHCl. (**C**) Representative immunoblots of detergent-insoluble, TL-resistant PrP species in brain homogenates from kiBVI^WT^ mice inoculated with either kiBVI^E200K^ (top) or kiBVI^D178N^ (bottom) brain extract treated with the indicated concentrations of GdnHCl. (**D**) Quantification of residual detergent-insoluble, TL-resistant PrP levels (mean ± SD) in brain homogenates from kiBVI^WT^ mice inoculated with brain extract from either kiBVI^E200K^ (red, *n* = 3) or kiBVI^D178N^ (blue, *n* = 3) mice following treatment with the indicated concentrations of GdnHCl. In **A** and **C**, PrP was detected using the antibody HuM-D13. In **B** and **D**, the calculated [GdnHCl]_50_ values are shown.

To characterize the conformational properties of the prions formed spontaneously in the brains of kiBVI^E200K^ and kiBVI^D178N^ mice, we used a conformational stability assay that measures the relative resistance of protein aggregates to denaturation with guanidine hydrochloride (Supplemental Figure 11) (65). This assay is commonly used to discriminate between different prion strains (66, 67). There was no difference in the conformational stability of the TL-resistant PrP aggregates in the brains of spontaneously ill kiBVI^E200K^ and kiBVI^D178N^ mice (Figure 7, A and B). Similarly, no differences in conformational stability were observed following transmission to kiBVI^WT^ mice (Figure 7, C and D).

## Discussion

Here, we demonstrate that kiBVI^D178N^ and kiBVI^E200K^ mice recapitulate many features of human prion disease without requiring the use of PrP overexpression, a non-native promoter, or injection with a preexisting source of PrP^Sc^. The mice developed progressive signs of neurological illness, prion disease–specific neuropathological changes such as spongiform degeneration and astrocytic gliosis, and TL- and PK-resistant PrP species in their brains. Interestingly, despite lower steady-state levels of PrP expression in the kiBVI^D178N^ line, the age of spontaneous disease onset was similar for the kiBVI^D178N^ and kiBVI^E200K^ lines, and TL-resistant PrP was more prevalent in kiBVI^D178N^ mice than in kiBVI^E200K^ mice. This may potentially be explained by a higher misfolding propensity conferred by the D178N mutation, perhaps related to the decreased stability of D178N-mutant BVPrP(I109) (53). Importantly, brain extracts from spontaneously sick mice expressing mutant BVPrP(I109) exhibited prion seeding activity and transmitted disease to kiBVI^WT^ mice, confirming the generation of authentic prion infectivity in the kiBVI^D178N^ and kiBVI^E200K^ lines. A limitation of our study is that while the D178N and E200K mutations are usually heterozygous in cases of genetic prion disease (i.e., WT and mutant PrP are simultaneously present), we used mice that were homozygous for the mutations and thus expressed higher relative levels of mutant PrP. However, it should be noted that individuals with homozygous E200K mutations have been identified and develop typical fCJD but at a younger age than those with a heterozygous mutation (68, 69).

The fact that kiBVI^D178N^ and kiBVI^E200K^ mice develop spontaneous neurological illness without requiring PrP overexpression makes them attractive models for interrogating the biological mechanisms governing spontaneous prion formation in the brain during genetic prion disease as well as uncovering therapeutic strategies for counteracting these processes. Small molecules known to interfere with the templated propagation of certain classical PrP^Sc^ strains do not prolong the onset of spontaneous disease in either the kiBVI^D178N^ or kiBVI^E200K^ lines, suggesting that inhibiting spontaneous prion formation may require a distinct therapeutic approach (70). To this end, the kiBVI^D178N^ and kiBVI^E200K^ lines will be particularly useful for assessing gene editing or PrP^C^-lowering treatments for spontaneous prion disease, which would be challenging in transgenic models with artificially high *PRNP* gene dosage. However, as with other animal models of prion disease (71, 72), assessing therapeutic efficacy in kiBVI^D178N^ and kiBVI^E200K^ lines will be challenging. In particular, the incomplete penetrance of spontaneous disease by 600 days of age and the variability in age of disease onset create logistical issues when designing therapeutic studies. Thus, these knockin models may be best employed when testing a drug with a known mechanism of action or when performing confirmatory studies on drugs that have already proven efficacious in a faster prion disease model, such as RML prion–inoculated mice.

We used the I109 polymorphic variant of BVPrP for our studies because it more readily forms prions spontaneously when overexpressed in transgenic mice than the M109 polymorphic variant (52). Indeed, transgenic mice that display modest overexpression of sheep PrP with isoleucine at position 112, which is analogous to position 109 in BVPrP, also develop a spontaneous and transmissible prion disease (73). Transgenic mice that overexpress other WT PrPs, including BVPrP(M109), can develop a proteinopathy that resembles prion disease but is non-transmissible (56, 74, 75). Thus, the presence of isoleucine at codon 109 of BVPrP may be critical for permitting the formation of PrP assemblies that are able to self-propagate. However, we did not observe any signs of spontaneous prion formation in aged kiBVI^WT^ mice. Therefore, PrP overexpression is required to initiate spontaneous prion formation in mice expressing WT PrP, even when a permissive substrate such as BVPrP is used. Besides I109, the sequence determinants of BVPrP that allow it to function as a permissive substrate for studying spontaneous prion formation remain incompletely defined. BVPrP-specific C-terminal residues (E227 and S230) seem to influence templated and spontaneous PrP misfolding, as do asparagine residues 155 and 170 within the α-helical domain (39, 56, 76–78). How any of these specific residue differences may promote spontaneous prion formation is unclear, since the structure of bank vole PrP^C^ is very similar to that of PrP^C^ from other mammals, other than the existence of a more rigid loop immediately prior to the second α-helix (79). The presence of a rigid loop in the structure of PrP^C^ has been linked to the appearance of spontaneous prion generation in certain lines of transgenic mice (80, 81).

**Figure 8:**
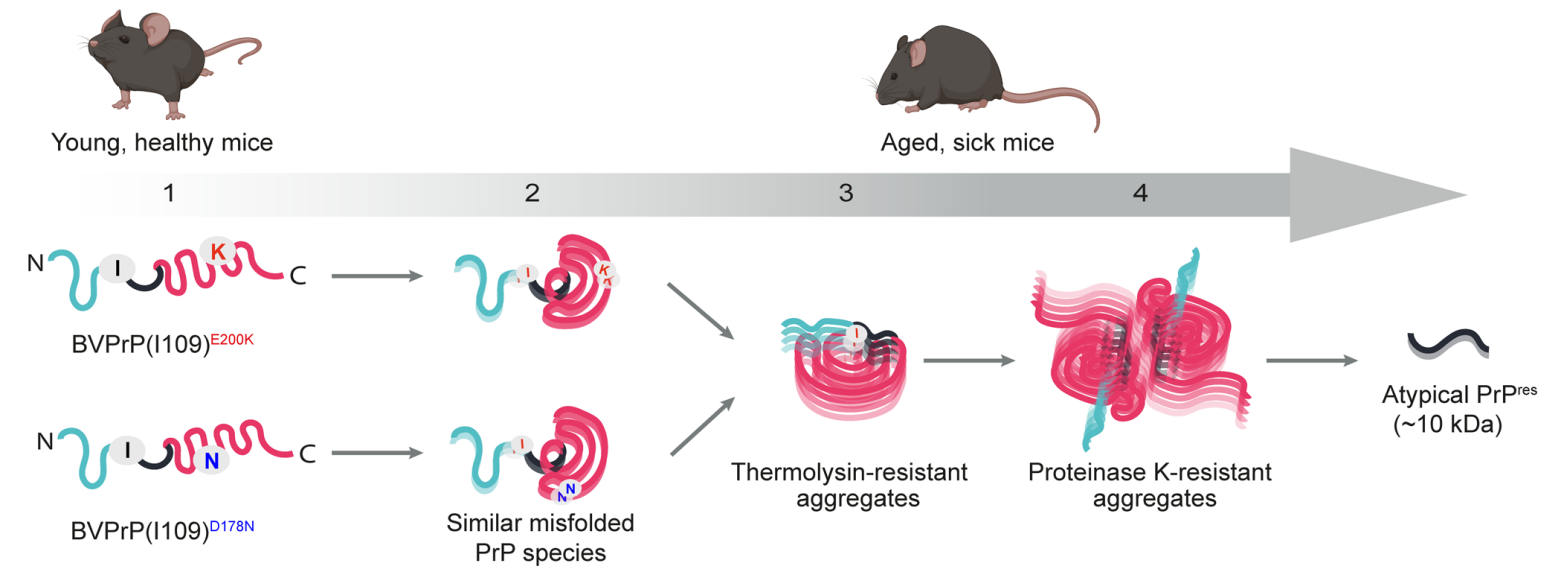
Model for the spontaneous generation of prions in kiBVI^E200K^ and kiBVI^D178N^ mice. (1.) Properly folded PrP is present in the brains of young, healthy knockin mice expressing mutant BVPrP(I109). (2.) As the mice age, the E200K and D178N mutations promote the generation of an identical or very similar misfolded BVPrP species. (3.) Polymerization of this misfolded species results in BVPrP aggregates that are resistant to thermolysin digestion and the appearance of clinical signs of neurological illness in the knockin mice. (4.) As the disease progresses, the BVPrP aggregates undergo conformational maturation to produce aggregates that contain a core region that is resistant to proteinase K digestion, producing an atypical PrP^res^ fragment of approximately 10 kDa. This PrP^res^ fragment does not contain either the D178N- or E200K-mutant residues.

While brains from most aged knockin mice expressing mutant BVPrP exhibited TL-resistant PrP, a smaller fraction contained PK-resistant PrP. This suggests that a spectrum of PrP assemblies develops in the knockin mice, with the formation of TL-resistant species likely preceding the generation of potentially larger or more densely packed PK-resistant aggregates (Figure 8). Whereas FFI and fCJD patients develop classical PrP^res^ with a molecular weight of 19–21 kDa following removal of N-linked glycans (11, 82), the brains of spontaneously sick kiBVI^D178N^ and kiBVI^E200K^ mice exhibited an atypical PK-resistant PrP fragment with a molecular weight of about 10 kDa, which is more reminiscent of those found in GSS (14–16). Short PK-resistant PrP fragments can also be found in Nor98/atypical scrapie in sheep and variably protease-sensitive prionopathy in humans (83–85). Interestingly, these atypical prion diseases, all of which are thought to result from spontaneous misfolding of PrP, can be efficiently transmitted to bank voles with the I109 polymorphism (37, 38, 86), and 7-kDa non-fibrillar PrP^res^ species purified from GSS brains are sufficient for disease transmission (87). Thus, the presence of the I109 residue may selectively stabilize these misfolded PrP species, allowing them to propagate and spread within the brain.

Potential relationships between atypical PrP^res^ and the classical PrP^res^ species found in most animal and human prion diseases remain to be fully investigated. Repeated passage of Nor98/atypical scrapie in transgenic mice expressing bovine PrP leads to a molecular phenotype indistinguishable from bovine spongiform encephalopathy, which is typified by the presence of classical PrP^res^ (88). Moreover, whereas I109 bank voles faithfully propagate the atypical short PrP^res^ fragments upon transmission of Nor98 scrapie, Nor98-inoculated M109 bank voles exhibit classical PrP^res^ (86). Intriguingly, cases of GSS caused by the P102L mutation exhibit an approximately 8-kDa PrP^res^ fragment, either alone or in combination with classical PrP^res^ (89, 90). Therefore, classical PrP^res^ may emerge from atypical PrP^res^, potentially due to the selection of rare conformational species within a mixture or deformed templating (91, 92).

The striking similarities in the neuropathological profiles, PrP^res^ signatures, conformational stabilities, behavior in the RT-QuIC assay, and transmission properties of the prions present in kiBVI^D178N^ and kiBVI^E200K^ mice imply that both lines spontaneously develop a highly similar, if not identical, atypical prion strain. This was unexpected given that transgenic models expressing E200K- or D178N-mutant BVPrP(I109) exhibit mutation-specific pathological signatures, although this could potentially be an artifact of PrP overexpression or differences in spatial expression patterns (53). Despite the absence of robust clinical illness and PrP^res^, MoPrP-based knockin models of the E200K and D178N mutations also exhibit different pathological phenotypes, perhaps since the sequence of MoPrP contains leucine at the residue corresponding to I109 of BVPrP (30, 31). However, translatome studies have shown that the molecular phenotypes in the MoPrP-based E200K and D178N knockin lines are more similar than expected (93). Although we cannot rule out a scenario in which the properties of BVPrP(I109) override mutation-specific strain generation, our data raise the possibility that the D178N and E200K mutations may uniformly act to promote the initial misfolding of PrP into a similar self-propagating species (Figure 8). The key properties of this initial misfolded species are likely determined by its atypical PK-resistant core rather than by the specific mutation, since similar PrP fragments are known to be both N- and C-terminally truncated and do not contain either residue 178 or 200 (9, 83, 87). The mechanism by which the D178N and E200K mutations promote the accumulation of misfolded BVPrP remains to be established. The mutations may directly promote spontaneous misfolding of BVPrP, stabilize the formation of prion assemblies, and/or reduce the clearance rates of aggregates.

In humans, disease-causing mutations within PrP clearly influence prion strain generation in the brain, even though a given mutation can specify formation of multiple prion strains (13). We propose a dual-hit model in which PrP mutations play 2 roles in genetic prion disease. First, they act by promoting the accumulation of an atypical misfolded PrP species; and second, later in the disease course, they bias the emergence of classical PrP^res^ toward conformations that are compatible with the specific mutation. Given that this latter phase might require a rare secondary misfolding event, our 2-phase model can provide insights into why genetic prion diseases manifest later in life, even though the mutations exist from birth. Facilitated by the inherent misfolding propensity of BVPrP and the capacity of I109 to stabilize atypical PrP^Sc^, kiBVI^D178N^ and kiBVI^E200K^ mice may effectively recapitulate pivotal early PrP misfolding events that occur in the brain during genetic prion disease.

## Methods

### Sex as a biological variable.

Our study examined male and female animals, and similar findings are reported for both sexes.

### Generation and characterization of knockin mice.

Gene targeting in V6.5 embryonic stem cells was performed at the DZNE/Bonn University using CRISPR/Cas9 as described previously (55). Plasmids containing the open reading frames (ORFs) of either WT, D178N-mutant, or E200K-mutant BVPrP(I109) were used as a starting point (53). Targeting constructs were generated by ligation of the respective variants of the BVPrP ORF between EagI and ClaI sites of the intermediate vector pWJPrP101 (55) containing homology regions and a neomycin selection cassette removable by Flp recombinase. The Cas9 vector used for double-strand break generation in the *Prnp* gene is available from Addgene (plasmid 78621) (55). Expansion of gene-edited embryonic stem cells and aggregation with diploid CD-1(ICR) mouse embryos were performed at The Centre for Phenogenomics (Toronto, Canada). Chimeric mice were identified by their mixed coat colors and then bred with B6(Cg)-*Tyr^c-2J^*/J mice (“B6-albino mice,” The Jackson Laboratory 000058) to identify those that underwent germline transmission events. Chimeras were crossed with a Flp deleter strain [B6.129S4-*Gt(ROSA)26Sor^tm1(FLP1)Dym^*/RainJ, The Jackson Laboratory 009086] to remove the selection cassette and then backcrossed with WT C57BL/6 mice to remove the Flp transgene. Mice that were positive for the BVPrP knockin allele and negative for the Flp transgene were then intercrossed to create homozygous BVPrP-knockin mice. All knockin lines were maintained by crossing of homozygous female with homozygous male mice.

Mice were housed at 4–5 animals per cage and were maintained on a 12-hour light/12-hour dark cycle, while being given unlimited access to food and water. Mice were assessed 3 times per week up to approximately 600 days of age for the presence of spontaneous signs of neurological illness consistent with experimental prion disease in rodents, including visible weight loss, aggression, ataxia, head bobbing or tilting, convulsion, circling behavior, kyphosis, limb abnormalities (paralysis, clasping, and/or reduced grip strength), loss of righting reflex, tail rigidity, bradykinesia, blank stare, and tremor. After 2 or more symptoms became apparent, mice were assessed daily and then euthanized upon progression to end-stage symptoms that included overt weight loss (~20% of body weight) accompanied by a reduced ability to ambulate and obtain food or water. Disease progression typically occurred over a period of 3–4 weeks. Some mice were euthanized before the onset of prominent weight loss because of extreme overgrooming/dermatitis in the neck or scalp area. Mice that were found dead in their cage in the absence of prior symptoms or were euthanized because of age-related intercurrent illness were excluded from the study (Supplemental Table 2). The brains of spontaneously ill mice, mice that remained asymptomatic at the experimental endpoint, or mice that were euthanized at defined ages were divided parasagitally and then either snap-frozen using dry ice and stored at –80°C or immersed in 10% neutral-buffered formalin for fixation. Mice were not perfused before brain collection.

### Brain homogenization and detergent extraction.

Frozen mouse hemibrains from BVPrP-knockin mice, non-transgenic C57BL/6 mice, or PrP^–/–^ mice (94) were homogenized using a Minilys bead beater (Precellys) to generate 10% (wt/vol) brain homogenates in Dulbecco’s phosphate-buffered saline (DPBS). For the preparation of detergent-extracted protein samples, 9 parts of 10% brain homogenate were mixed with 1 part of 10× detergent extraction buffer (5% [wt/vol] sodium deoxycholate and 5% [vol/vol] NP-40 prepared in DPBS). The samples were incubated on ice for 20 minutes with vortexing every 5 minutes and then centrifuged at 5,000*g* for 5 minutes at 4°C. The protein concentration in the supernatant was determined using the bicinchoninic acid (BCA) assay (Thermo Fisher Scientific 23227).

### Prion transmission assays.

Groups of 7–9 kiBVI^WT^ mice at approximately 6 weeks of age were anesthetized using isoflurane gas and then intracerebrally inoculated with 30 μL of 1% brain homogenate prepared from frozen hemibrains and diluted in PBS containing 5% (wt/vol) BSA. Inoculations were performed into the right cerebral hemisphere to a depth of approximately 3 mm using a tuberculin syringe with an attached 27-gauge, 0.5-inch needle (BD Biosciences 305945). Inoculated mice were housed in groups of 3–4 animals in disposable cages and monitored for the development of neurological illness as described above. Once the inoculated mice reached disease end stage, they were euthanized, and then their brains were removed and divided into hemispheres using the sagittal plane. Alternatively, the brains of asymptomatic mice were collected at the experimental endpoint (540 days after inoculation). The left hemisphere was frozen and stored at –80°C, while the right hemisphere was fixed in 10% neutral-buffered formalin and stored at 22°C (room temperature) for neuropathological examination. Mice were not perfused before brain collection. All inoculation experiments used roughly equal numbers of male and female mice, except for 1 experiment involving inoculation of brain homogenate from an asymptomatic 20-month-old kiBVI^WT^ mouse, which used only female mice.

### Detergent insolubility assays.

Detergent-extracted brain homogenates containing 50–100 μg total protein were diluted in 1× detergent extraction buffer (0.5% [wt/vol] sodium deoxycholate, 0.5% [vol/vol] NP-40 prepared in DPBS) and then ultracentrifuged at 100,000*g* for 1 hour at 4°C using a TLA-55 rotor (Beckman). The supernatant was removed, and the pellet was resuspended in 1× Bolt LDS sample buffer (Thermo Fisher Scientific B0007) containing 2.5% (vol/vol) β-mercaptoethanol. The samples were boiled at 95°C for 10 minutes and then analyzed by immunoblotting.

### Enzymatic digestions.

PNGase F digestions (New England Biolabs P0704S) were performed according to the manufacturer’s recommendation. Briefly, 50 μg of detergent-extracted brain homogenate was mixed with 5 μL of Glycoprotein Denaturing Buffer (10×) and incubated at 95°C for 10 minutes. The samples were allowed to cool down on ice, and then 5 μL each of 10% NP-40 and GlycoBuffer 2 as well as 0.5 μL of PNGase F were added to make a final reaction volume of 50 μL. After incubation at 37°C for 1–2 hours, the reaction was stopped by addition of LDS sample buffer (1× final concentration) and incubation of the samples at 95°C for 10 minutes. Samples were then analyzed by immunoblotting.

Thermolysin (TL) was purchased from MilliporeSigma (T7902) and dissolved in dH_2_O to generate a stock concentration of 1 mg/mL. For TL digestions, 500 μg of detergent-extracted brain homogenate was diluted into a final volume of 100 μL 1× detergent extraction buffer containing 100 μg/mL TL for a final TL/protein ratio of 1:50. The reaction mixture was incubated in a thermomixer at 37°C for 1 hour with 600 rpm shaking, and digestions were stopped by the addition of EDTA to a final concentration of 5 mM. Sarkosyl was then added to a final concentration of 2% (vol/vol). This was followed by ultracentrifugation at 100,000*g* for 1 hour at 4°C. Finally, the supernatant was gently removed, and the pellet was resuspended in 1× Bolt LDS sample buffer containing 2.5% (vol/vol) β-mercaptoethanol, boiled, and analyzed by immunoblotting. Alternatively, detergent-extracted brain homogenate was treated with different concentrations of TL at 37°C for 1 hour and then analyzed directly by immunoblotting without isolation of the insoluble fraction. For PK digestions, a similar protocol to that used for TL digestions was used, except that 1 mg of detergent-extracted brain homogenate was digested with 50 μg/μL PK (Thermo Fisher Scientific EO0491) in a volume of 400 μL for a final PK/protein ratio of 1:50, and the reaction was stopped by addition of PMSF to a final concentration of 2 mM.

### Immunoblotting.

Proteins were separated on 10% Bolt Bis-Tris gels (Thermo Fisher NW00100BOX or NW00102BOX) and then transferred onto 0.45 mm Immobilon-P polyvinylidene fluoride membranes (MilliporeSigma IPVH00010) using Tris-glycine transfer buffer containing 20% (vol/vol) methanol. Membranes were blocked in 5% (wt/vol) skim milk prepared in 1× Tris-buffered saline containing 0.05% (vol/vol) Tween-20 (TBST) overnight at 4°C or for at least 1 hour at 22°C. The following day, membranes were incubated with primary antibody for 1 hour at 22°C. The primary antibodies that were used include anti-PrP antibodies HuM-D18 (1:5,000 dilution) (95), HuM-P (1:10,000 dilution) (96), HuM-D13 (1:10,000 dilution) (95), POM1 (MilliporeSigma MABN2285; 1:5,000 dilution), HuM-R1 (1:10,000 dilution) (95), EP1802Y (Abcam ab52604; 1:10,000 dilution), and SAF-32 (Cayman Chemical 189720; 1:5,000 dilution); and an anti-GFAP antibody (Thermo Fisher Scientific A-21282; 1:10,000 dilution). The HuM-D18 and HuM-P antibodies were produced in-house, whereas HuM-D13 and HuM-R1 were provided by Stanley Prusiner (University of California, San Francisco, California, USA). Blots were washed 3 times with TBST (10 minutes each), incubated with horseradish peroxidase–linked (HRP-linked) secondary antibodies (Bio-Rad 172-1011 or 172-1019 or Thermo Fisher Scientific 31414) diluted in blocking buffer for 1 hour at 22°C, and then washed an additional 3 times with TBST. Membranes were developed using Western Lightning ECL Pro (Revvity NEL122001EA) or SuperSignal West Dura Extended Duration Substrate (Thermo Fisher Scientific 34075), and the signal was detected by exposure to x-ray films or using the LI-COR Odyssey Fc system. For reprobing, blots were washed with TBST and then incubated in blocking buffer containing 0.05% (wt/vol) sodium azide overnight at 4°C to inactivate the HRP. The next day, blots were reprobed with anti-actin 20-33 antibody (MilliporeSigma A5060; 1:10,000 dilution).

### Enzyme-linked immunosorbent assays.

Relative BVPrP expression levels in the knockin mice were determined by enzyme-linked immunosorbent assay (ELISA). Various amounts of recombinant BVPrP(I109) (44) and detergent-extracted brain homogenates from kiBVI^WT^ mice were used as standards. Immulon 4 HBX 96-well plates (VWR 62402-959) were coated with the anti-PrP antibody HuM-D18 at 5 μg/mL in coating buffer (200 mM NaH_2_PO_4_, pH 7.5) overnight at 4°C. The plate was blocked with 1% BSA diluted in PBS with 0.05% Tween-20 (PBS-T) at 22°C for more than 2 hours, followed by washing with PBS-T. Thereafter, the standards and test samples prepared in PBS containing 0.5% Triton-X were added in triplicates, and the plate was incubated overnight at 4°C with shaking. After 4 washes with PBS-T, the HRP-labeled HuM-P detection antibody (provided by Stanley Prusiner) was added at 1:50,000 dilution in blocking buffer and incubated at 22°C for 2 hours with shaking. The plate was thoroughly washed 5 times with PBS-T, and 100 μL of TMB-Blue substrate (BioShop TMB333.100) was added followed by incubation in the dark for 5–10 minutes. The reaction was stopped by addition of 100 μL of 1 M HCl to each well. Finally, the absorbance at 450 nm was read using a BMG CLARIOstar microplate reader.

### RT-QuIC assays.

Recombinant BVPrP (residues 23–231, M109 isoform) was expressed and purified as described previously (44). Recombinant protein was dialyzed in 10 mM sodium phosphate buffer, pH 7.4 (Molecular Toxicology Inc., 26-588), overnight at 4°C and then ultracentrifuged at 100,000*g* at 4°C for 1 hour to remove any preformed aggregates. Seeds were prepared by dilution of 10% brain homogenates (“10^–1^ dilution”) from knockin mice or RML prion–inoculated C57BL/6 mice in 1× PBS with 0.05% SDS and 1× N2 supplement (Thermo Fisher Scientific 17502048). The RT-QuIC reaction mixture consisted of 0.1 mg/mL recombinant BVPrP; 10 mM sodium phosphate buffer, pH 7.4; 300 mM NaCl; 1 mM EDTA; and 10 μM thioflavin T. Reactions were carried out in triplicate in black, 96-well clear-bottom plates (Thermo Fisher Scientific 265301). Each well contained 98 μL of reaction mixture and 2 μL of diluted seed. The sealed plates were incubated at 42°C in a BMG CLARIOstar microplate reader with cycles of 1-minute shake (700 rpm double orbital) and 1-minute rest/read for about 70 hours. The fluorescence excitation and emission wavelengths were 444 ± 5 nm and 485 ± 5 nm, respectively, with a gain setting of 1,600. Lag phases were calculated as previously described (97). Plateau fluorescence values were calculated as the mean of the readings within the final hour of the assay.

### Conformational stability assays.

Assays were performed using a concentration gradient of guanidine hydrochloride (GdnHCl). Twenty microliters of detergent-extracted brain homogenates was added to an equal volume of 2× GdnHCl stocks to create final concentrations of 1, 1.5, 2, 2.5, 3, 3.5, and 4 M GdnHCl. To generate the 0 M sample, an equal volume of DPBS was added. The samples were incubated at 22°C for 2 hours with shaking (800 rpm). The concentration of GdnHCl was then diluted to 0.4 M by addition of detergent extraction buffer (1% final concentration) and DPBS. The samples were then subjected to TL digestion (100 μg/mL) as described above and ultracentrifuged at 100,000*g* for 1 hour at 4°C. The supernatants were gently removed, and the pellets were resuspended in 1× Bolt LDS sample buffer containing 2.5% (vol/vol) β-mercaptoethanol and boiled for 10 minutes at 95°C. Densitometry analysis was carried out using ImageJ (NIH) from 3 independent replicates, and [GdnHCl]_50_ values (the concentration of GdnHCl at which 50% of the PrP aggregates are solubilized) were calculated using a variable slope (4-parameter) dose-response model in GraphPad Prism as described previously (65).

### Neuropathology.

Formalin-fixed hemibrains were processed using a Leica Pearl automated tissue processor and then embedded in paraffin. Sections (5 μm) were cut, mounted on positively charged glass slides, and then dried overnight at 37°C. Slides were deparaffinized using xylene, rehydrated through a graded series of ethanol, and then either stained with hematoxylin and eosin (H&E) or processed for immunohistochemistry. For H&E staining, slides were stained with Hematoxylin 560 MX (Leica 3801575) for 2 minutes, rinsed with dH_2_O, incubated with Blue Buffer 8 (Leica 3802915) for 90 seconds, rinsed with dH_2_O, and then incubated with Define MX-AQ (Leica 3803595) for 30–45 seconds. After rinsing with dH_2_O, the slides were incubated with Eosin 515 LT (Leica 3801619) for 2 minutes, washed 3 times with 100% ethanol (3 minutes each), incubated with 3 changes of xylenes (5 minutes each), and then coverslipped and mounted with Permount (Fisher Scientific SP15-100). For GFAP immunostaining, the Polink-2 Plus HRP Rabbit DAB Detection kit (GBI Labs) was used. Slides were treated with 3% (vol/vol) hydrogen peroxide for 10 minutes and then washed with dH_2_O. Antigen retrieval was performed using 10 mM sodium citrate, pH 6, for 30 minutes at 95°C, and then slides were cooled and washed twice (2 minutes each) with TBST. The rabbit polyclonal GFAP antibody (Dako Z0334; 1:4,000 dilution) was applied overnight at 4°C. After rinsing with TBST, development, and counterstaining with hematoxylin, slides were dehydrated and then coverslipped and mounted using Permount. For PrP immunohistochemistry, slides were treated with 98% formic acid for 10–15 minutes, rinsed 3 times with dH_2_O (5 minutes each), and then processed using the M.O.M. kit (Vector Laboratories). Slides were incubated with the mouse monoclonal PrP antibody 9A2 (Wageningen Bioveterinary Research; 1:500 dilution) for 30 minutes at 22°C. Slides were developed using the NovaRed system (Vector Laboratories), counterstained with hematoxylin, dehydrated, and then coverslipped and mounted using VectaMount. All slides were digitized using the Zeiss Axio Scan.Z1 slide scanner, and then representative images were captured using PMA.start (Pathomation).

For quantification of vacuolation, snapshots of scanned slides from H&E-stained tissue were taken and then converted to 8-bit black and white images using ImageJ. Freehand regions of interest were drawn around the desired brain region, and then the threshold was set to 0–210 to reveal areas of the brain without stain. Following conversion to binary and creation of a binary mask, the Analyze Particles function in ImageJ was used to determine the percentage brain area covered by vacuolation. A size range of 8 to infinity and a circularity of 0.8 to 1.0 were used to ensure that the interiors of cerebral blood vessels were not counted as vacuoles.

For quantification of astrocytic gliosis, snapshots of scanned slides from GFAP-stained tissue were taken and converted to 8-bit black and white images using ImageJ. The H-DAB model in the IHC Toolbox plug-in in ImageJ was used to remove non-stained regions. Freehand regions of interest were then drawn around the desired brain region, and the threshold was set to 0–100. The percentage area covered by GFAP staining was then measured.

### Statistics.

All statistical analysis was conducted using GraphPad Prism (version 10.0.0) with a significance threshold of *P* less than 0.05. No tests for data normality were performed. Survival curves were compared using the log-rank (Mantel-Cox) test. Sex-specific differences in the age of spontaneous disease onset as well as RT-QuIC parameters were analyzed by the Mann-Whitney test. ELISA measurements were compared using Welch’s ANOVA followed by Dunnett’s T3 multiple-comparison test. Detergent-insoluble PrP levels were compared using 1-way ANOVA followed by Tukey’s multiple-comparison test. Neuropathological indicators were compared using a Kruskal-Wallis test followed by Dunn’s multiple-comparison test.

### Study approval.

All mouse experiments were performed in accordance with the guidelines set by the Canadian Council on Animal Care under protocols (AUP 4263.17 and 6322.3) approved by the University Health Network Animal Care Committee (Toronto, Ontario, Canada).

### Data availability.

All data generated or analyzed during this study are included in this published article and in the Supporting Data Values file.

## Author contributions

All authors contributed to the study’s conception and design. Material preparation and data collection and analysis were performed by SM, MECB, LK, ES, HA, JKG, KLF, DJW, SS, SAB, WSJ, and JCW. The first draft of the manuscript was written by SM, MECB, and JCW, and all authors commented on previous versions of the manuscript. All authors read and approved the final manuscript.

## Supplementary Material

Supplemental data

Unedited blot and gel images

Supporting data values

## Version 1 08/01/2024

Electronic publication
