# Supplementary material for: Convergent generation of atypical prions in knockin mouse models of genetic prion disease
Source: J Clin Invest. 2024 Aug 1;134(15):e176344. doi: 10.1172/JCI176344 (PMC11291267; doi:10.1172/JCI176344)
Supplement: Supplemental data [file jci-134-176344-s234.pdf]

Supplemental Information: Convergent generation of atypical prions in knock-in mouse models of genetic prion disease

Surabhi Mehra[1], Matthew E.C. Bourkas[1,2], Lech Kaczmarczyk[3,4], Erica Stuart[1], Hamza Arshad[1,2], Jennifer K. Griffin[1], Kathy L. Frost[5], Daniel J. Walsh[6], Surachai Supattapone[6,7], Stephanie A. Booth[5,8], Walker S. Jackson[3,4], and Joel C. Watts[1,2,§]

[1]Tanz Centre for Research in Neurodegenerative Diseases, University of Toronto, Toronto, Ontario, Canada

[2]Department of Biochemistry, University of Toronto, Toronto, Ontario, Canada

[3]Wallenberg Center for Molecular Medicine, Department of Biomedical and Clinical Sciences, Linköping University, Linköping, Sweden

[4]German Center for Neurodegenerative Diseases (DZNE), Bonn, Germany

[5]One Health Division, National Microbiology Laboratory, Public Health Agency of Canada, Winnipeg, Manitoba, Canada

[6]Department of Biochemistry and Cell Biology, Geisel School of Medicine at Dartmouth, Hanover, New Hampshire, USA

[7]Department of Medicine, Geisel School of Medicine at Dartmouth, Hanover, New Hampshire, USA

[8]Department of Medical Microbiology and Infectious Diseases, Faculty of Health Sciences, University of Manitoba, Winnipeg, Manitoba, Canada

[§]To whom correspondence should be addressed at: Krembil Discovery Tower, Rm. 4KD481, 60 Leonard Ave., Toronto, ON, Canada, M5T 0S8; Tel: (416) 507-6891; Fax: (416) 603-6435; joel.watts@utoronto.ca

Supplemental Table 1. Spontaneous neurological illness in knock-in mice expressing mutant BVPrP

Line	Mean age of disease onset ± SD (d)	Signs of neurological illness (n/n_0)	Mice showing TL-resistant PrP (n/n_0)	Mice showing PK-resistant PrP (n/n_0)
$kiBVI^{WT}$	> 598-603	0/22	0/21	0/2
$kiBVI^{E200K}$	524 ± 56	14/23	14/22	2/6
$kiBVI^{D178N}$	537 ± 57	15/23	22/22	7/11

n, number of mice; n_0, number of mice examined

Supplemental Table 2. List of mice removed from the study due to intercurrent illness

Mouse line	Animal ID	Sex	Age (d)	Notes	TL-resistant PrP?
kiBVI^{WT}	3162	F	583	Found dead in cage (previously asymptomatic)	Brain not collected
	3405	F	458	Found dead in cage (previously asymptomatic)	Brain not collected
	4015	F	544	Found dead in cage (previously asymptomatic)	Brain not collected
	4019	M	520	Euthanized due to difficulty breathing	No
	4589	F	588	Found dead in cage (previously asymptomatic)	Brain not collected
	5152	F	515	Euthanized due to liver tumor and corneal ulcer	No
kiBVI^{E200K}	3798	F	514	Found dead in cage (previously asymptomatic)	Brain not collected
	3799	F	540	Euthanized due to corneal ulcer	No
	3824	F	533	Found dead in cage (previously asymptomatic)	Brain not collected
	3826	F	502	Euthanized due to enlarged spleen	No
	3831	M	547	Euthanized due to fighting injuries	No
	3917	M	539	Euthanized due to fighting injuries	No
kiBVI^{D178N}	3391	M	562	Found dead in cage (previously asymptomatic)	Brain not collected

Supplemental Table 3. Transmission of brain homogenates from BVPrP knock-in mice to kiBVI^{WT} mice

Inoculum			Mean incubation period ± SD (d)	Signs of neurological illness (n/n_0)	Mice showing TL-resistant PrP (n/n_0)	Mice showing PK-resistant PrP (n/n_0)
Line	Age (d)	Spontaneously ill?				
kiBVI^{WT}	600	No	> 540	0/8	0/6	0/6
	602	No	> 540	0/9	0/5	0/5
kiBVI^{E200K}	560	Yes	312 ± 36	8/8	8/8	8/8
	467	Yes	324 ± 64	8/8	8/8	8/8
kiBVI^{D178N}	491	Yes	248 ± 134	2/6	2/6*	2/6^
	522	Yes	354 ± 20	3/6	3/6*	2/6^

n, number of mice; n_0, number of mice examined

* 4 of the 5 brains from kiBVI^{WT} mice inoculated with kiBVI^{D178N} samples that showed TL-resistant PrP were from asymptomatic mice collected at 541 dpi

^ All of the brains from kiBVI^{WT} mice inoculated with kiBVI^{D178N} samples that showed PK-resistant PrP were from asymptomatic mice collected at 541 dpi

Supplemental Table 4. RT-QuIC on brain extracts from aged BVPrP knock-in mice

Mouse line	Age (d)	Symptomatic?	Positive replicates in RT-QuIC[1]
kiBVI^{WT}	600	-	0/3
	600	-	1/3
	602	-	0/3
	600	-	1/3
	603	-	0/3
	598	-	0/3
kiBVI^{E200K}	510	+	3/3
	467	+	3/3
	539	+	2/3
	594	+	3/3
	560	+	3/3
	441	+	2/3
kiBVI^{D178N}	522	+	3/3
	557	+	3/3
	576	+	3/3
	568	+	3/3
	577	+	3/3
	491	+	3/3

[1]Brain extracts were tested at a 10^{-4} dilution

Supplemental Table 5. RT-QuIC on brain extracts from inoculated kiBVI^{WT} mice

Inoculum	Days post-inoculation	Symptomatic?	Positive replicates in RT-QuIC[1]
kiBVI^{WT}	540	-	0/3
	540	-	1/3
	540	-	0/3
	540	-	0/3
	540	-	1/2
	540	-	1/3
kiBVI^{E200K}	315	+	3/3
	357	+	3/3
	341	+	3/3
	344	+	3/3
	315	+	3/3
	312	+	3/3
kiBVI^{D178N}	541	-	3/3
	541	-	3/3
	541	-	3/3
	541	-	3/3
	541	-	2/3
	541	-	0/3

[1]Brain extracts were tested at a 10^{-4} dilution

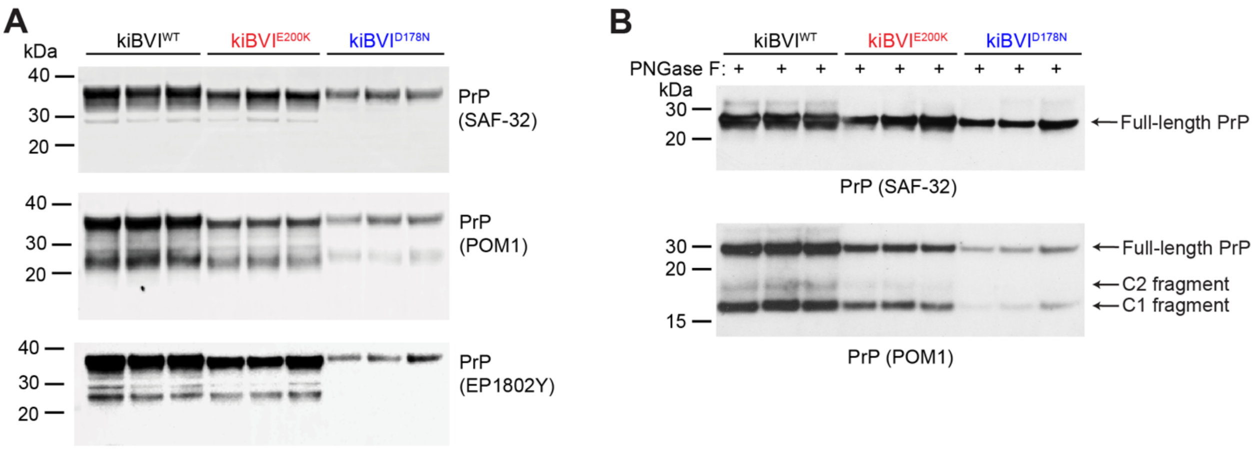

Supplemental Figure 1. Characterization of knock-in mice expressing wild-type or mutant bank vole PrP using additional antibodies. (**A**) Immunoblots for PrP in brain extracts from 3 mice each for the kiBVI^{WT}, kiBVI^{E200K}, and kiBVI^{D178N} lines. BVPrP was detected using the antibodies SAF-32, POM1, and EP1802Y. The SAF-32 and POM1 immunoblots were respectively generated by reprobing the HuM-P and HuM-D18 immunoblots shown in Figure 1B following treatment of the membranes with 0.05% (w/v) sodium azide to inactivate the original HRP signal. (**B**) Immunoblots for PrP in PNGase F-treated brain extracts from 3 mice each for the kiBVI^{WT}, kiBVI^{E200K}, and kiBVI^{D178N} lines. BVPrP was detected using the antibodies SAF-32 and POM1. Full-length BVPrP as well as the C1 and C2 endoproteolytic products are indicated. The SAF-32 and POM1 immunoblots were respectively generated by reprobing the HuM-P and HuM-D18 immunoblots shown in Figure 1E following treatment of the membranes with 0.05% (w/v) sodium azide to inactivate the original HRP signal.

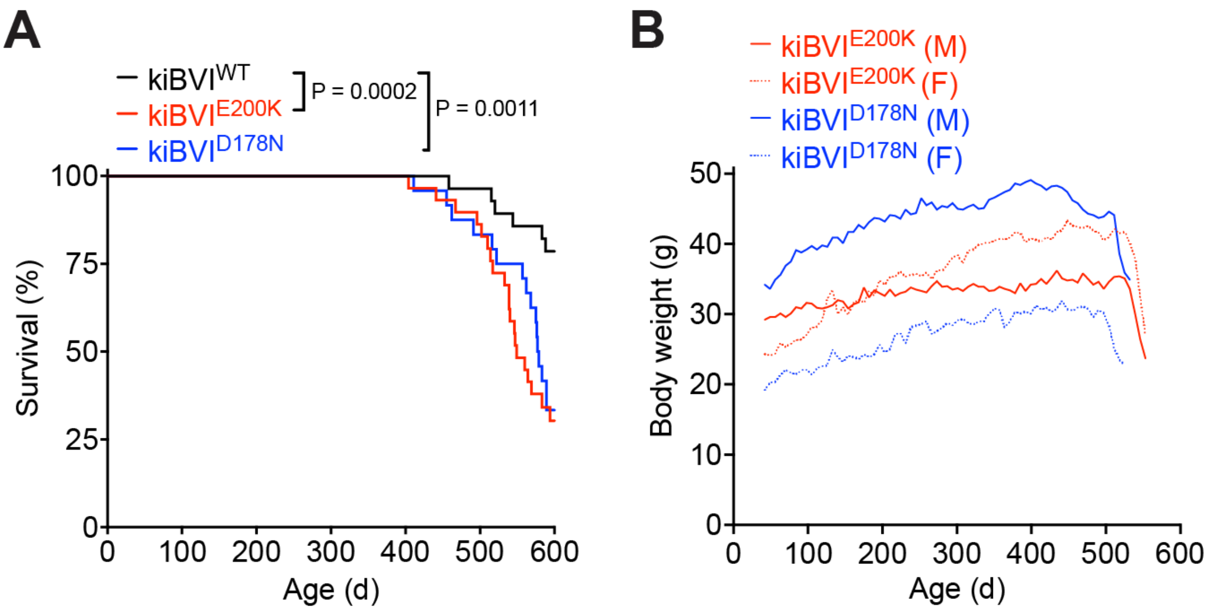

Supplemental Figure 2. Spontaneous disease in kiBVI^{E200K} and kiBVI^{D178N} mice. (**A**) All-cause mortality survival curves for kiBVI^{WT} (black, n = 28), kiBVI^{E200K} (red, n = 29), and kiBVI^{D178N} (blue, n = 24) mice. Mice that were found dead or were euthanized due to intercurrent illness are included. Statistical significance was determined using the Log-rank test. (**B**) Body weight trajectories for representative male (M) and female (F) kiBVI^{E200K} and kiBVI^{D178N} mice.

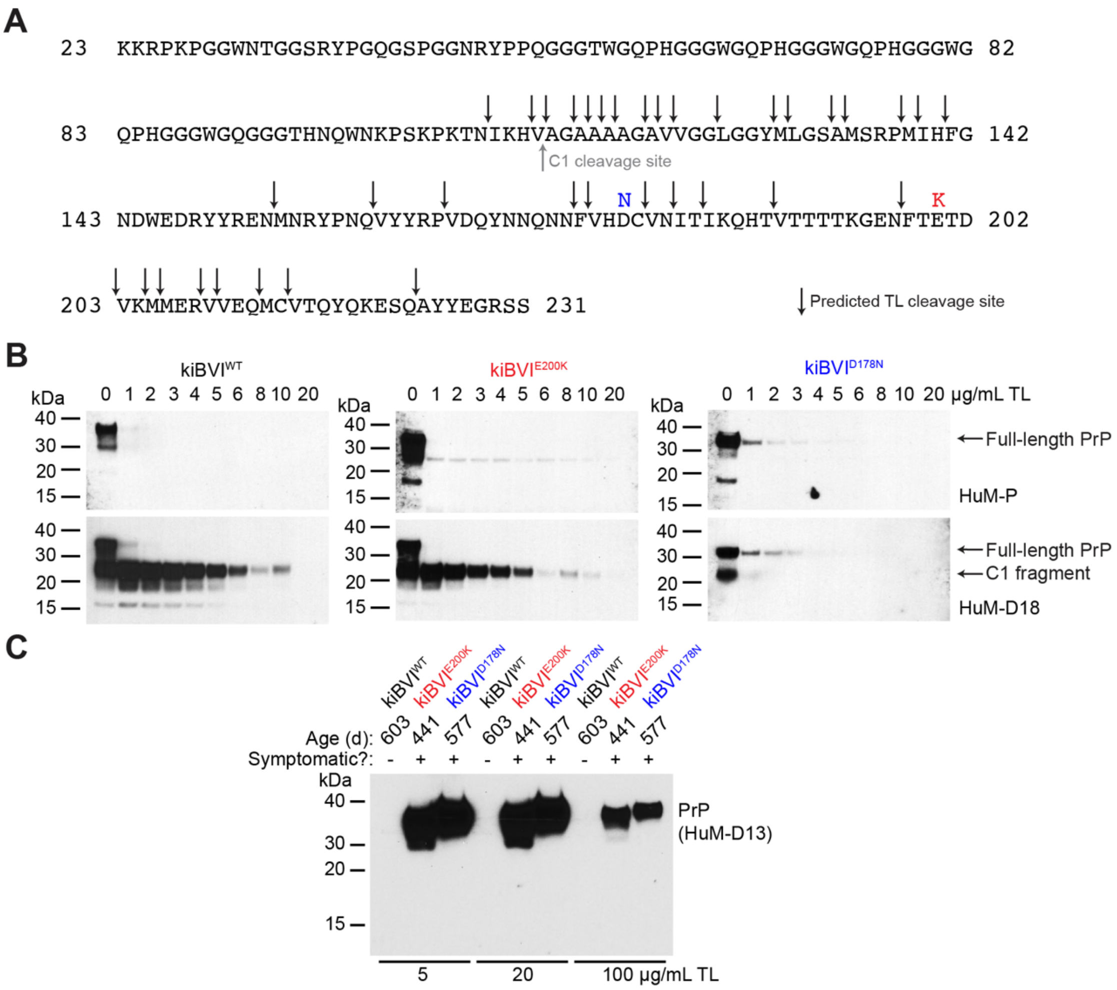

Supplemental Figure 3. Thermolysin digestion of brain extracts from young and aged knock-in mice. (**A**) Predicted thermolysin (TL) cleavage sites in the sequence of the mature form of BVPrP(I109) lacking the N- and C-terminal signal sequences. The locations of the D178N and E200K mutations as well as the C1 endoproteolytic cleavage site are also shown. (**B**) Immunoblots for PrP in brain extracts from young (3-month-old) kiBVI^{WT} (left), kiBVI^{E200K} (middle), and kiBVI^{D178N} (right) mice treated with the indicated concentrations of TL. Blots were probed with the antibodies HuM-P (top blots) or HuM-D18 (bottom blots). The location of full-length and C1 PrP species are indicated. (**C**) Immunoblot for detergent-insoluble PrP species in brain homogenates from asymptomatic 20-month-old kiBVI^{WT} mice and spontaneously ill kiBVI^{E200K} and kiBVI^{D178N} mice treated with the indicated concentrations of TL. PrP was detected using the antibody HuM-D13.

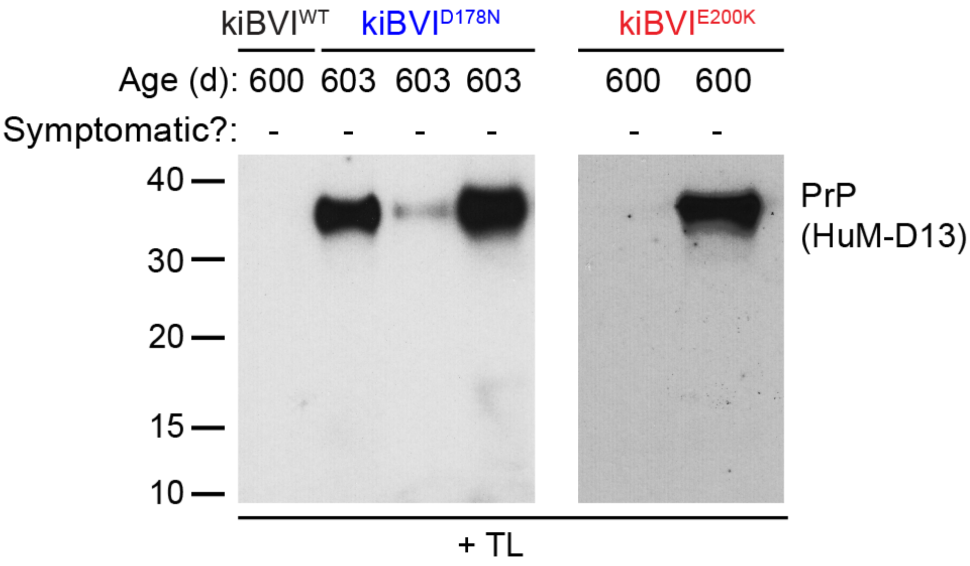

Supplemental Figure 4. Thermolysin-resistant PrP in aged, asymptomatic mice expressing mutant bank vole PrP. Immunoblots for detergent-insoluble, TL-resistant PrP species in brain extracts from asymptomatic, 20-month-old kiBVI^{WT}, kiBVI^{D178N}, and kiBVI^{E200K} mice. PrP was detected using the antibody HuM-D13. The molecular weight markers indicate kDa.

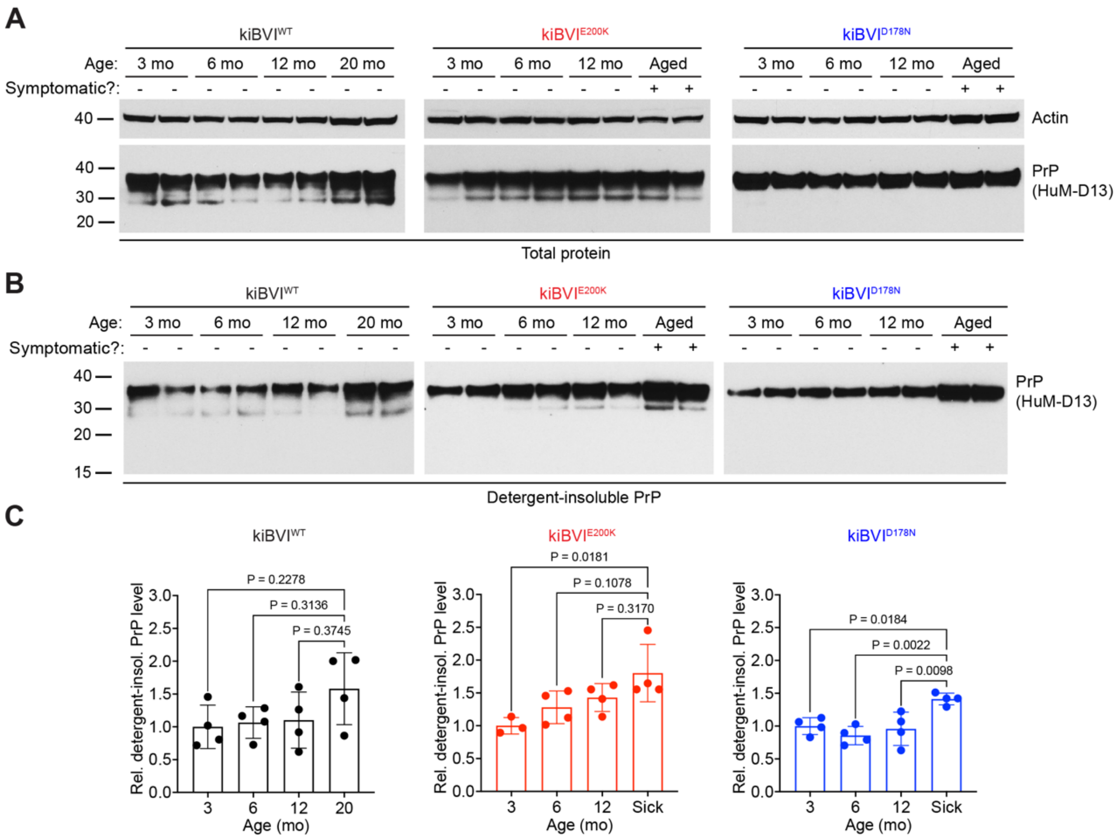

Supplemental Figure 5. Detergent-insoluble PrP levels in knock-in mice at different ages. (**A**) Immunoblots of total PrP levels in brain extracts from kiBVI^{WT} (left), kiBVI^{E200K} (middle), and kiBVI^{D178N} (right) mice at the indicated ages. Two independent mice per age were analyzed. For the kiBVI^{E200K} and kiBVI^{D178N} lines, aged mice with spontaneous neurological illness were also examined. (**B**) Immunoblots for detergent-insoluble PrP species in brain extracts from kiBVI^{WT} (left), kiBVI^{E200K} (middle), and kiBVI^{D178N} (right) mice at the indicated ages. Two independent mice per age were analyzed. PrP was detected using the antibody HuM-D13. Exposure times were adjusted for optimal visualization and do not reflect differences in detergent-insoluble PrP levels between the three knock-in lines. The molecular weight markers indicate kDa. (**C**) Quantification of detergent-insoluble PrP species (mean ± SD) in the three lines at various ages relative to the levels present in the brains of 3-month-old mice for each line (n = 3-4 mice per age). Statistical significance was assessed using one-way ANOVA followed by Tukey's multiple comparisons test.

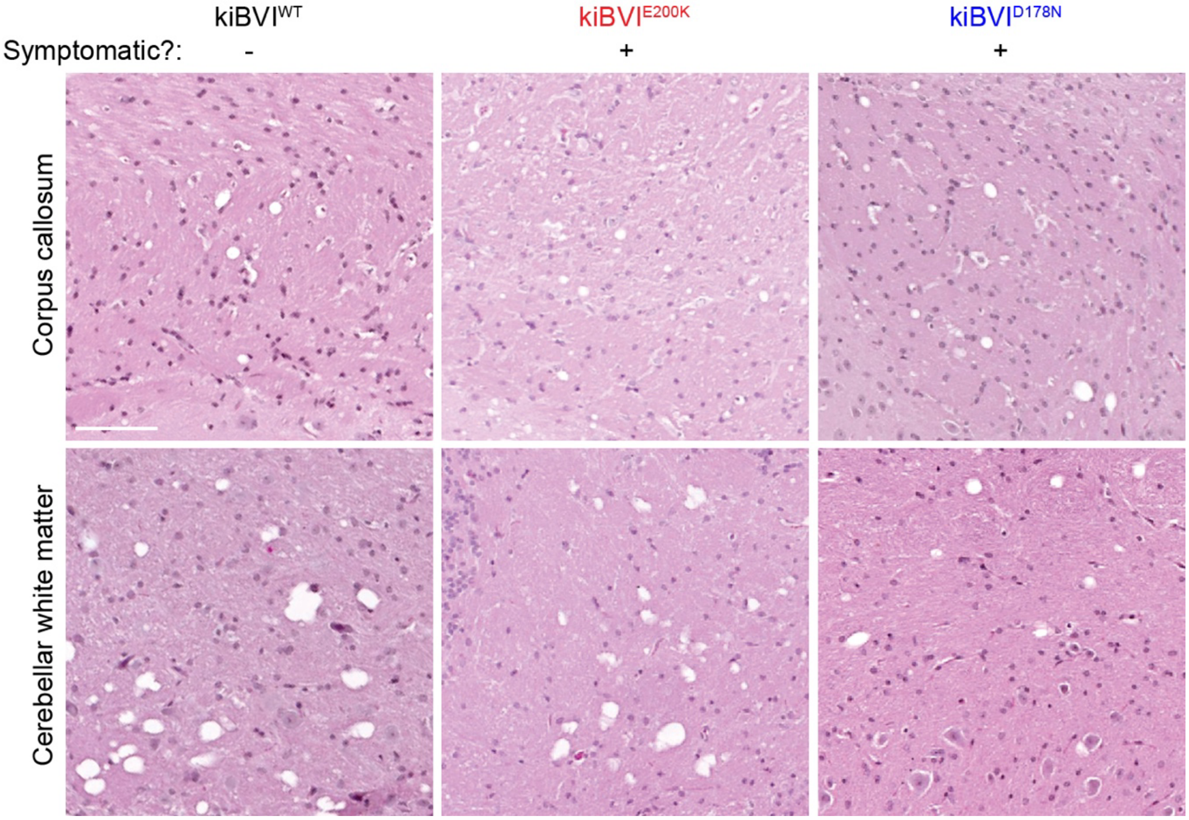

Supplemental Figure 6. White matter vacuolation in aged knock-in mice expressing wild-type or mutant bank vole PrP. Representative H&E-stained sections of the corpus callosum and cerebellar white matter from 20-month-old asymptomatic $kiBVI^{WT}$ mice as well as spontaneously ill $kiBVI^{E200K}$ and $kiBVI^{D178N}$ mice. Scale bar = 100 μm (applies to all sections).

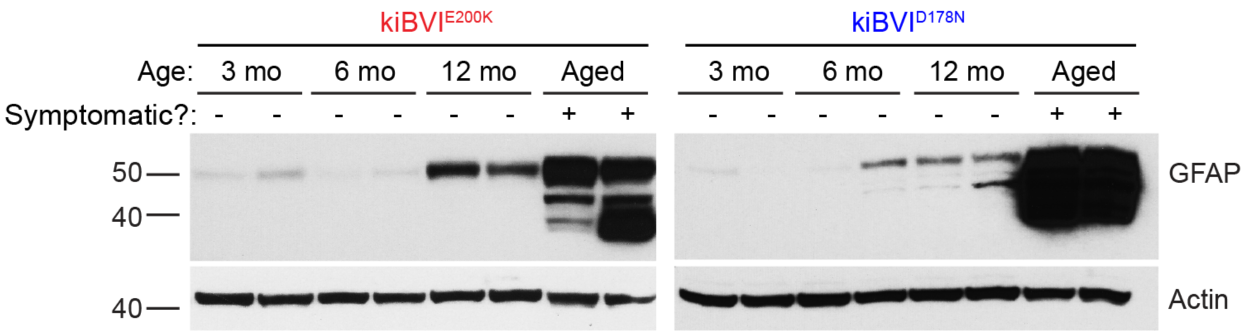

Supplemental Figure 7. Kinetics of GFAP accumulation in knock-in mice expressing mutant BVPrP. Immunoblots of GFAP levels in brain extracts from kiBVI^{E200K} (left) and kiBVI^{D178N} (right) mice at the indicated ages. Two independent mice per age were analyzed, and the blots were reprobed with an antibody against actin. The molecular weight markers indicate kDa.

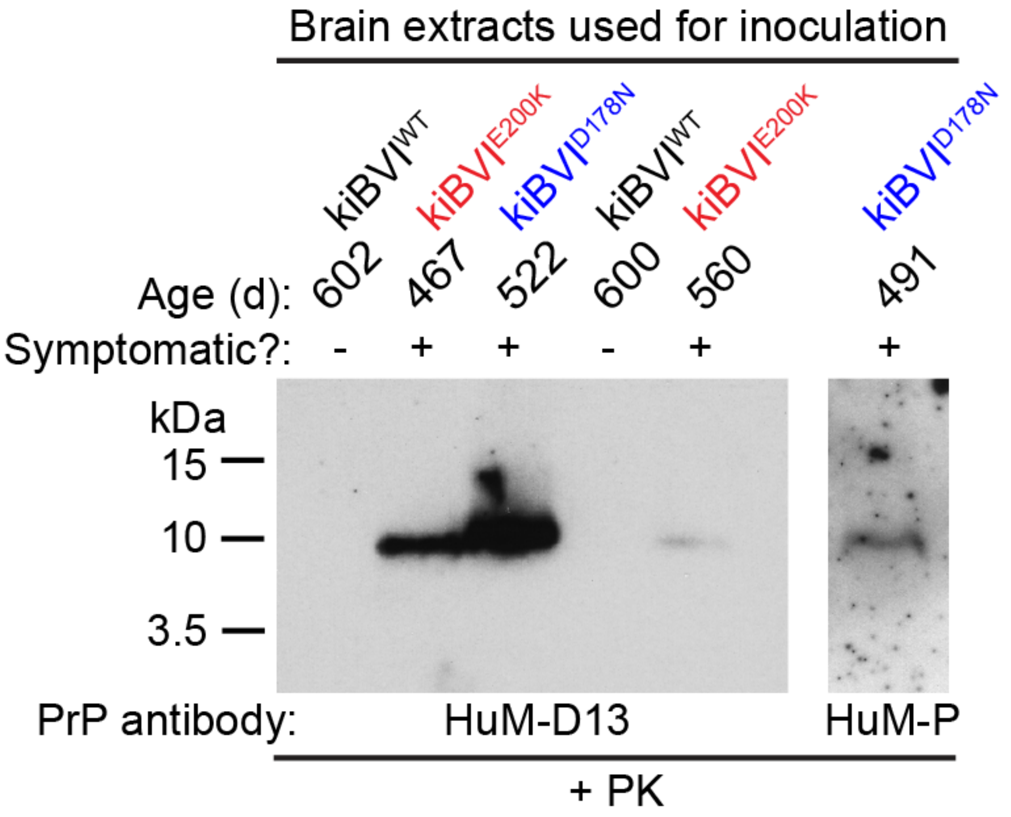

Supplemental Figure 8. PK-resistant PrP levels in brain extracts used for transmission studies. Immunoblots of detergent-insoluble PK-resistant PrP levels in the brain extracts from asymptomatic kiBVI^{WT} mice or spontaneously ill kiBVI^{E200K} and kiBVI^{D178N} mice used as inocula for transmission studies in kiBVI^{wt} mice. PrP was detected using the antibody HuM-D13 (left blot) or HuM-P (right blot).

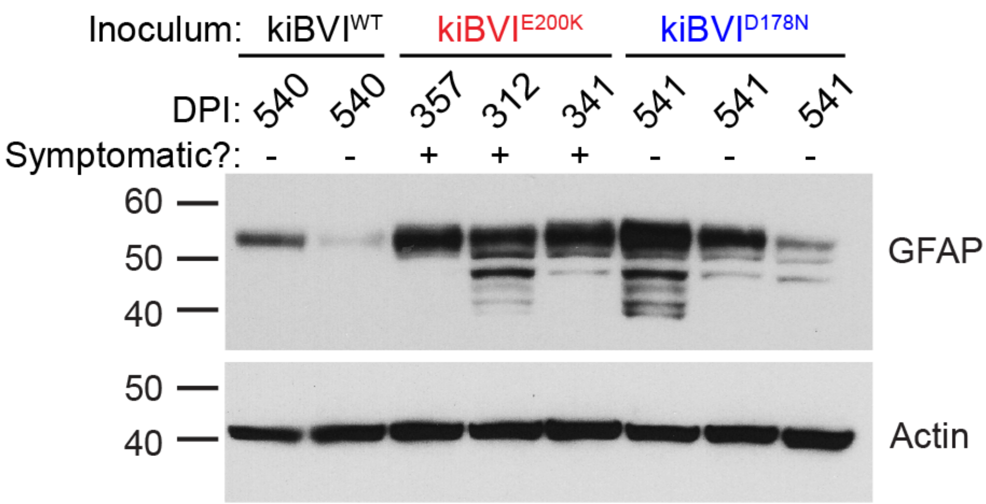

Supplemental Figure 9. GFAP levels in inoculated kiBVI^{WT} mice. Immunoblot of GFAP levels in brain extracts from kiBVI^{WT} mice at the indicated DPI with brain extract from asymptomatic kiBVI^{WT} mice, symptomatic kiBVI^{E200K} mice, or symptomatic kiBVI^{D178N} mice. The blot was reprobed with an antibody against actin. The molecular weight markers indicate kDa.

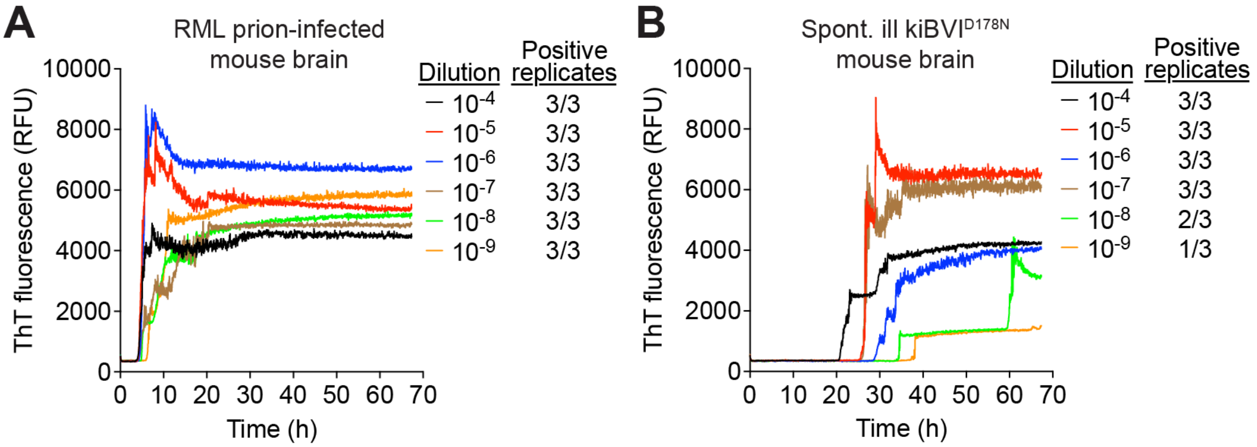

Supplemental Figure 10. Titration of prion seeding activity in the brains of an RML prion-inoculated and a spontaneously ill kiBVI^{D178N} mouse. RT-QuIC assays on brain extracts (ten-fold dilutions from 10^{-4} to 10^{-9}) from a terminally ill C57BL/6 mouse at 143 DPI with RML prions (**A**) or a spontaneously ill kiBVI^{D178N} mouse at 577 days of age (**B**). Three technical replicates were performed for each brain sample, and the number of positive replicates for each dilution is shown.

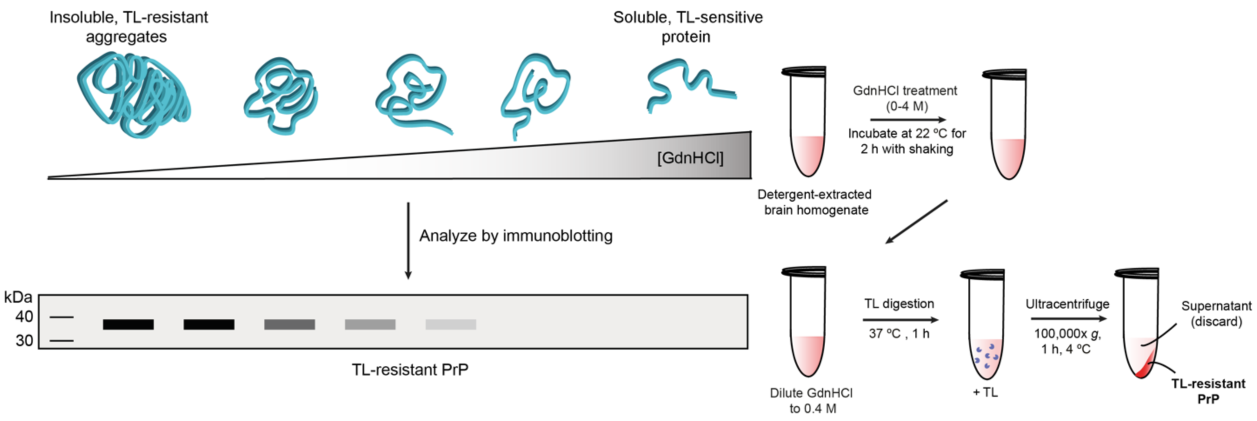

Supplemental Figure 11. Experimental schematic of the conformational stability assay. Following treatment with various concentrations of GdnHCl, samples are digested with TL and then the relative level of detergent-insoluble BVPrP species are quantified by immunoblotting.
