# Supplementary material for: Convergent generation of atypical prions in knockin mouse models of genetic prion disease
Source: J Clin Invest. 2024 Aug 1;134(15):e176344. doi: 10.1172/JCI176344 (PMC11291267; doi:10.1172/JCI176344)
Supplement: Unedited blot and gel images [file jci-134-176344-s235.pdf]

Full unedited blots for Figure 1

B

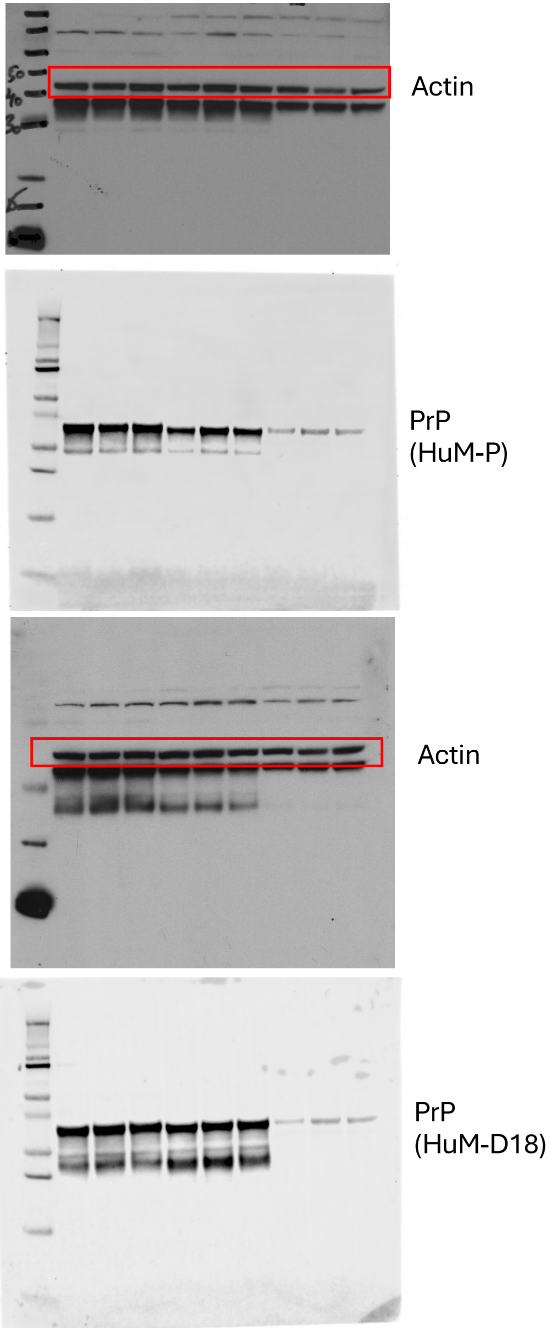

D

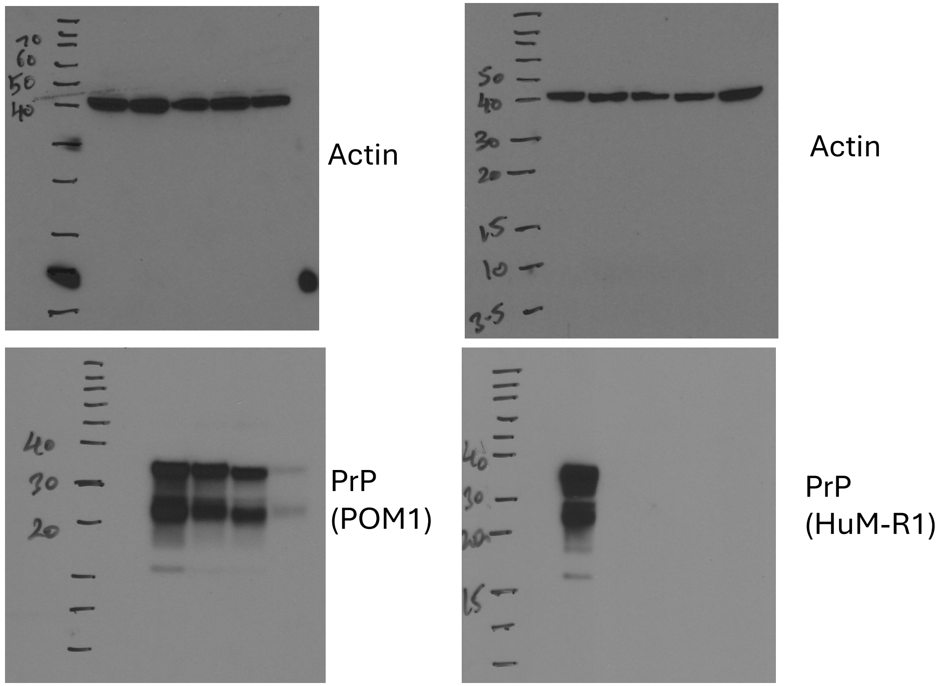

E

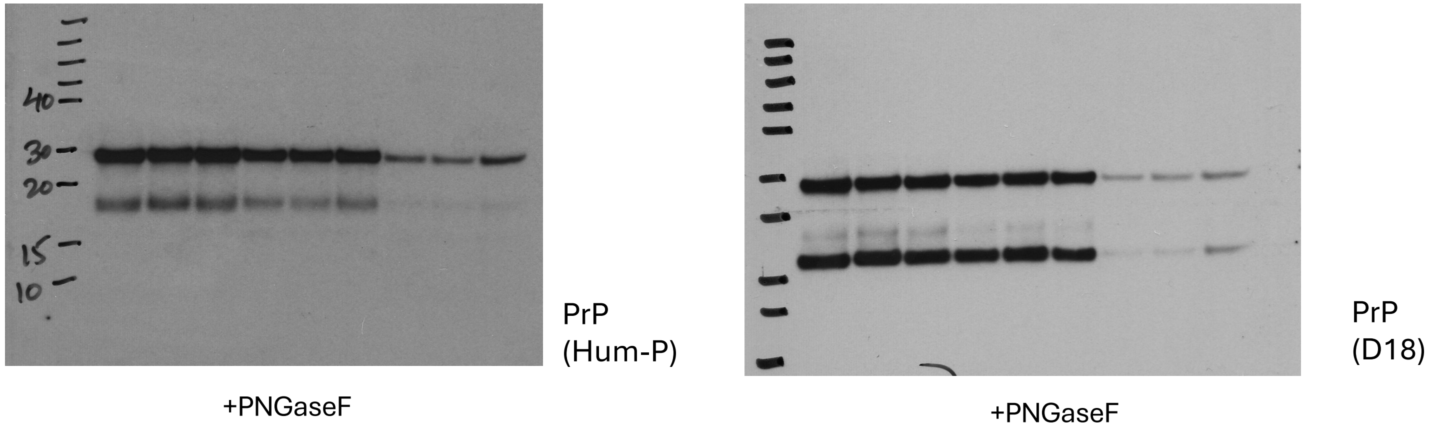

Full unedited blots for Figure 3

B

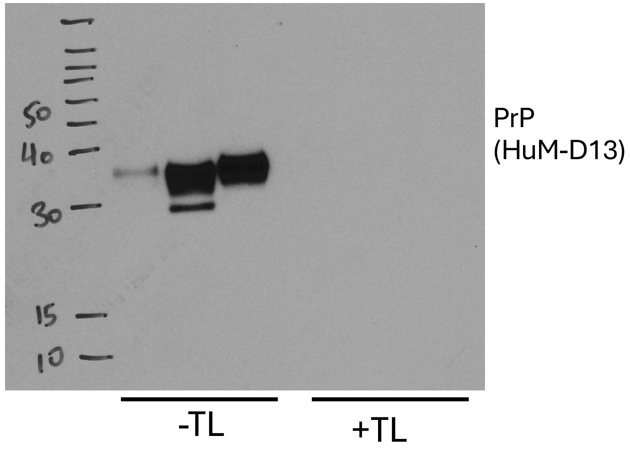

C

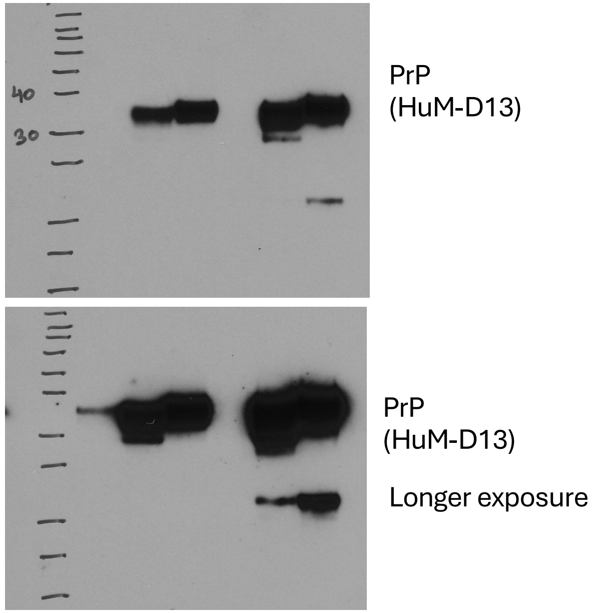

D

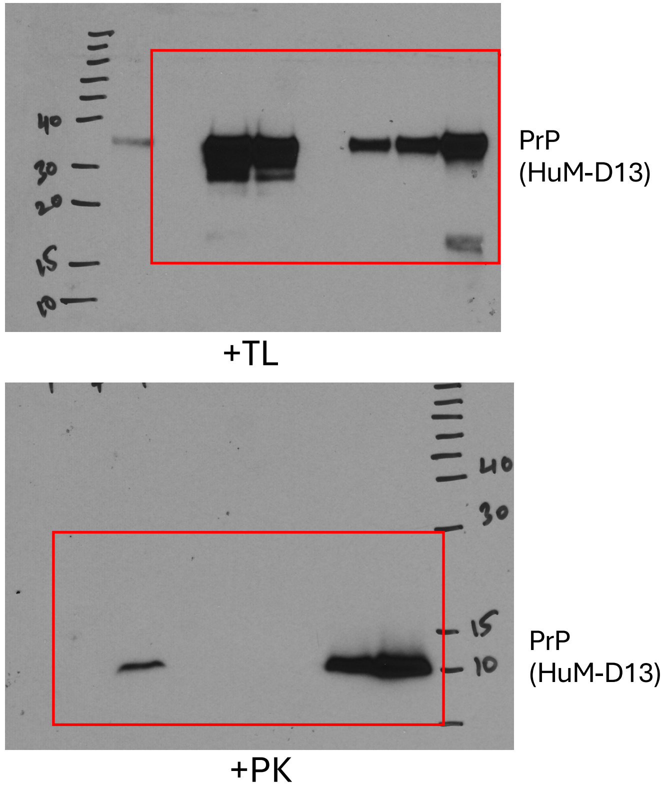

E

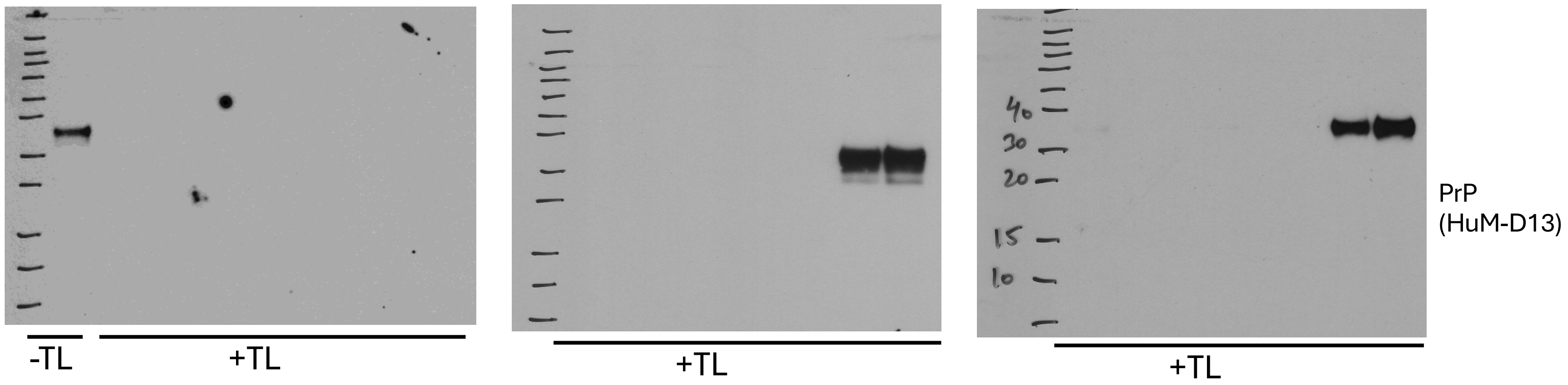

Full unedited blots for Figure 4

E

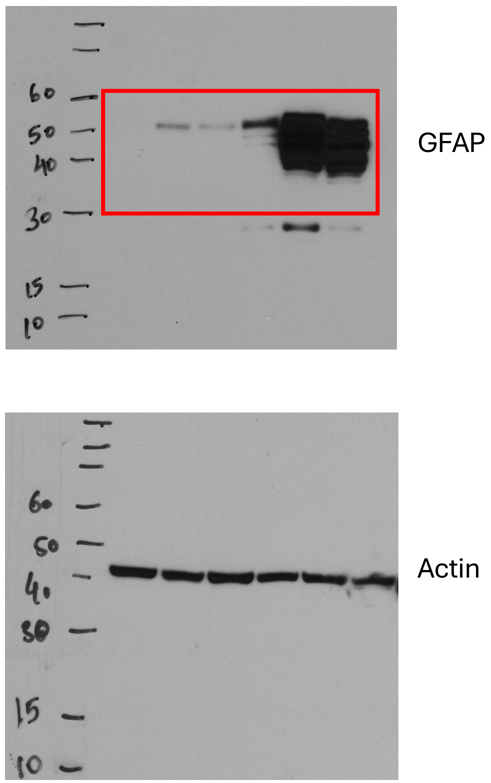

Full unedited blots for Figure 5

B

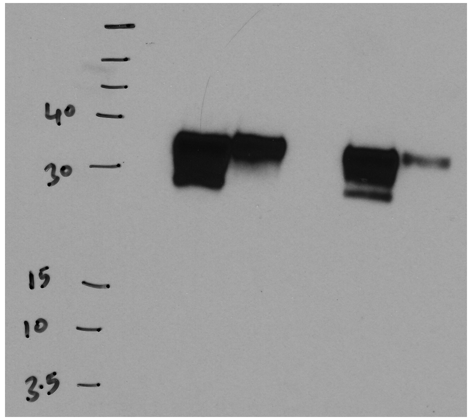

PrP
(HuM-D13)

+TL

F

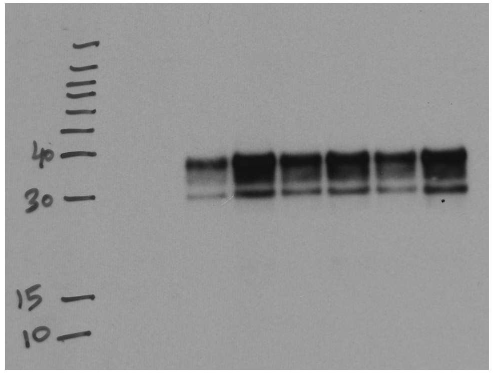

PrP
(HuM-D13)

+TL

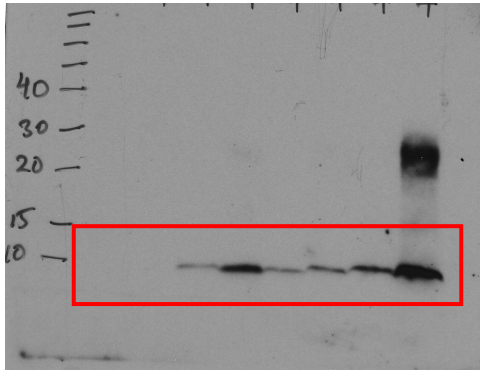

PrP
(HuM-D13)

+PK

Full unedited blots for Figure 7

A

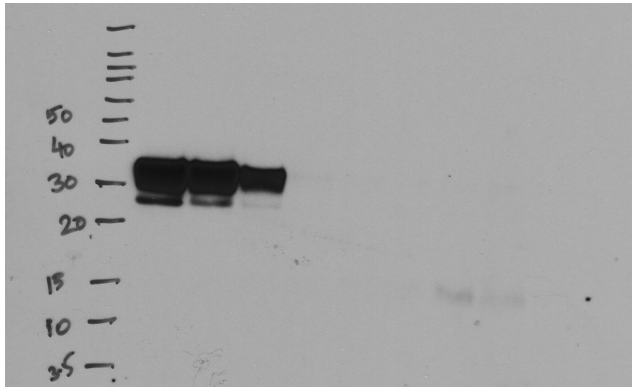

PrP
(HuM-D13)

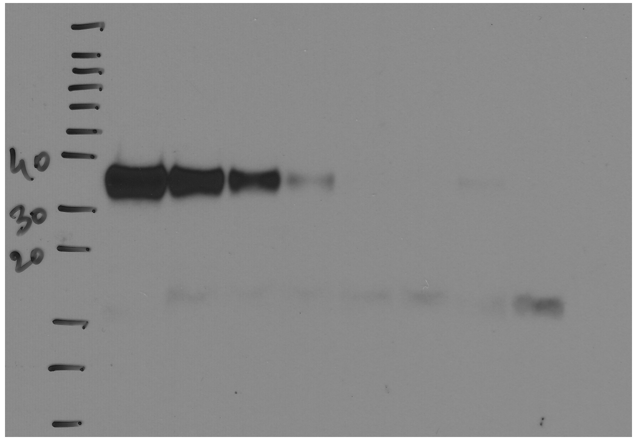

PrP
(HuM-D13)

C

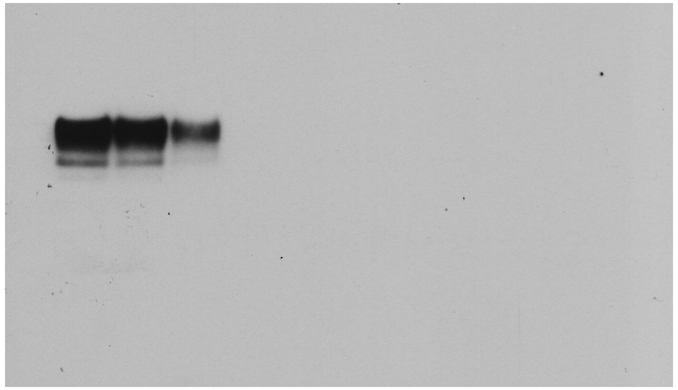

PrP
(HuM-D13)

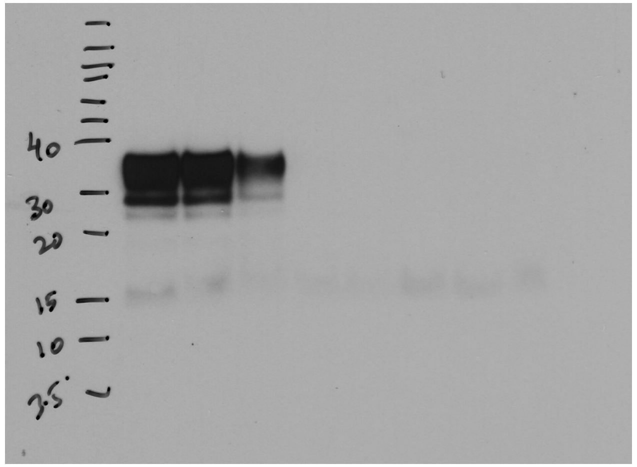

PrP
(HuM-D13)

Full unedited blots for Supplementary Figure 1

A

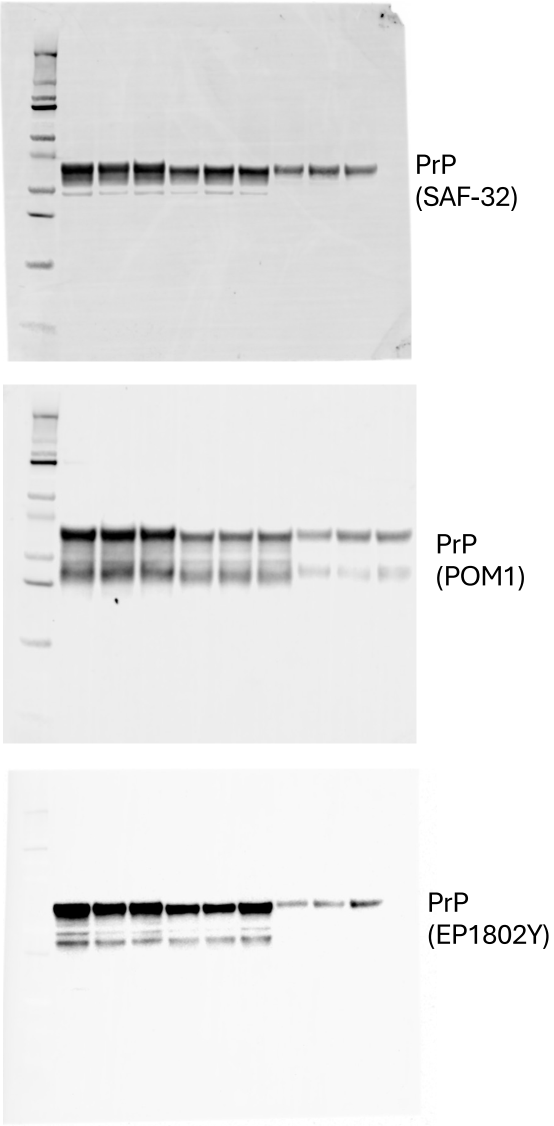

PrP
(SAF-32)

PrP
(POM1)

PrP
(EP1802Y)

B

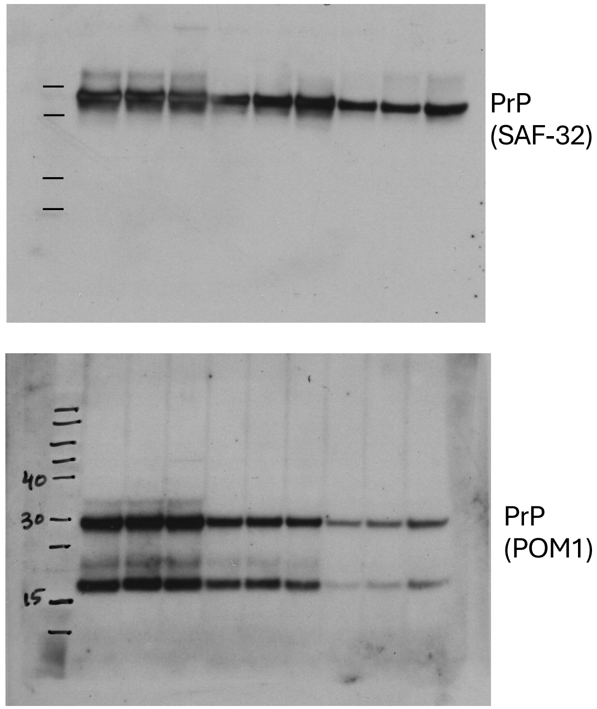

PrP
(SAF-32)

PrP
(POM1)

Full unedited blots for Supplementary Figure 3

B

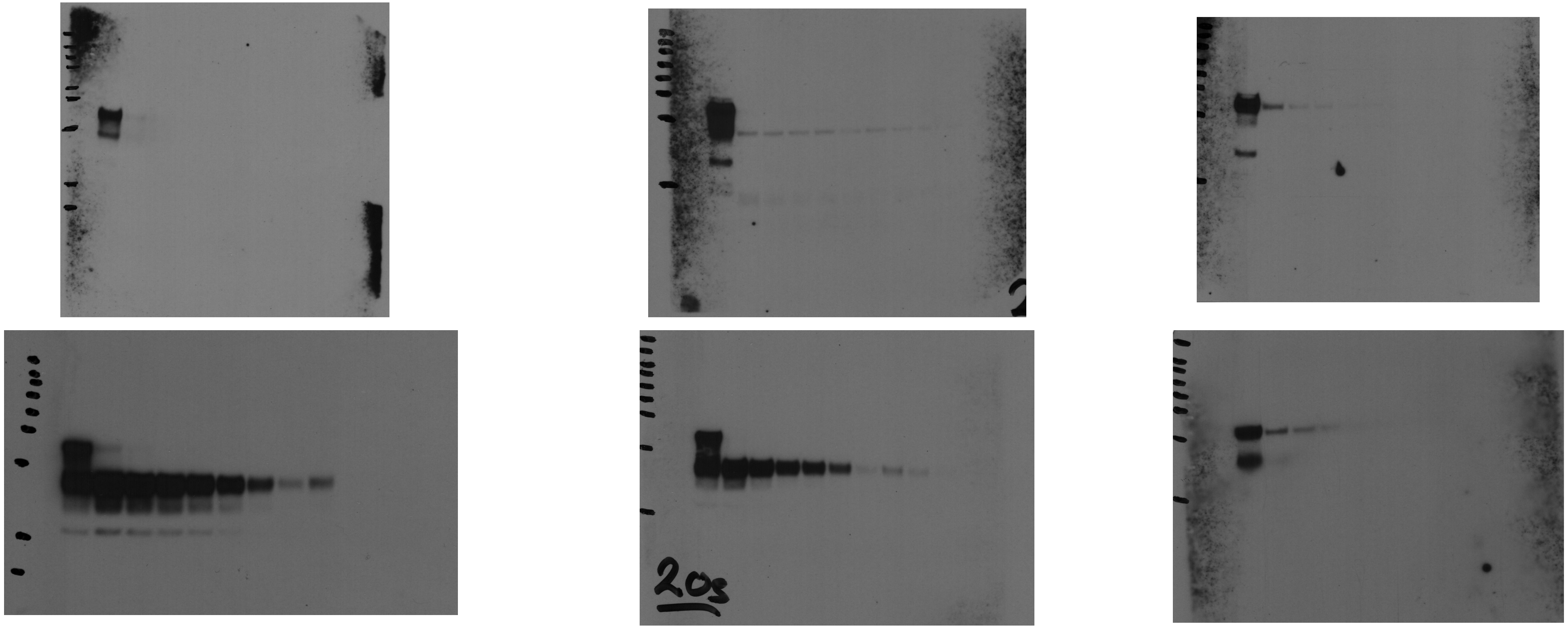

PrP (HuM-P)

PrP (HuM-D18)

kiBVI^{wt} kiBVI^{E200K} kiBVI^{D178N}

C

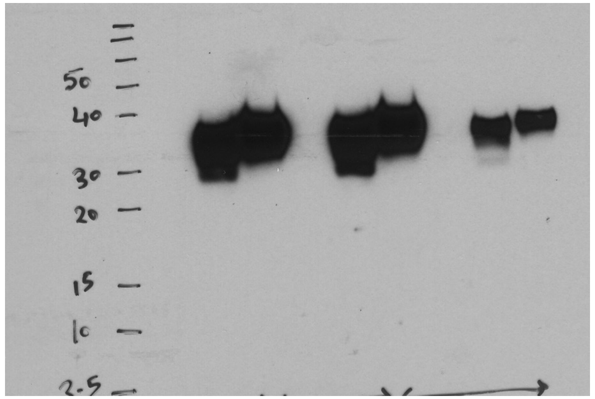

PrP (HuM-D13)

5 20 100 µg/mL TL

Full unedited blots for Supplementary Figure 4

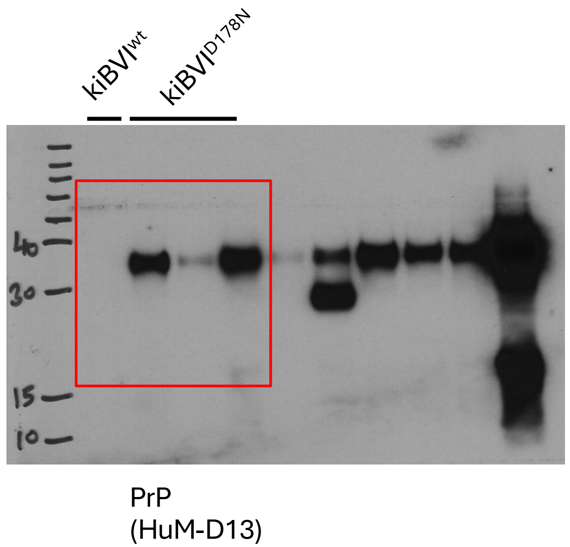

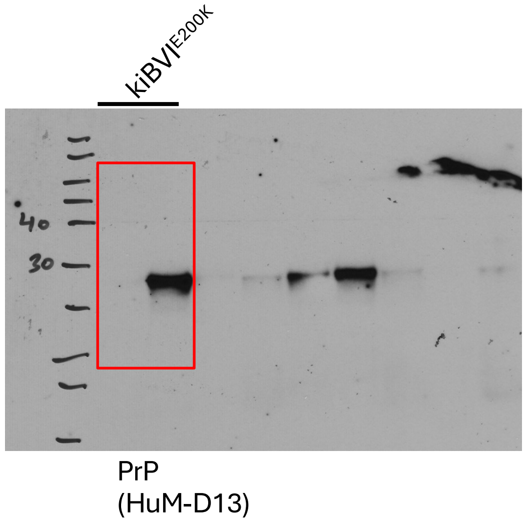

Full unedited blots for Supplementary Figure 5

A

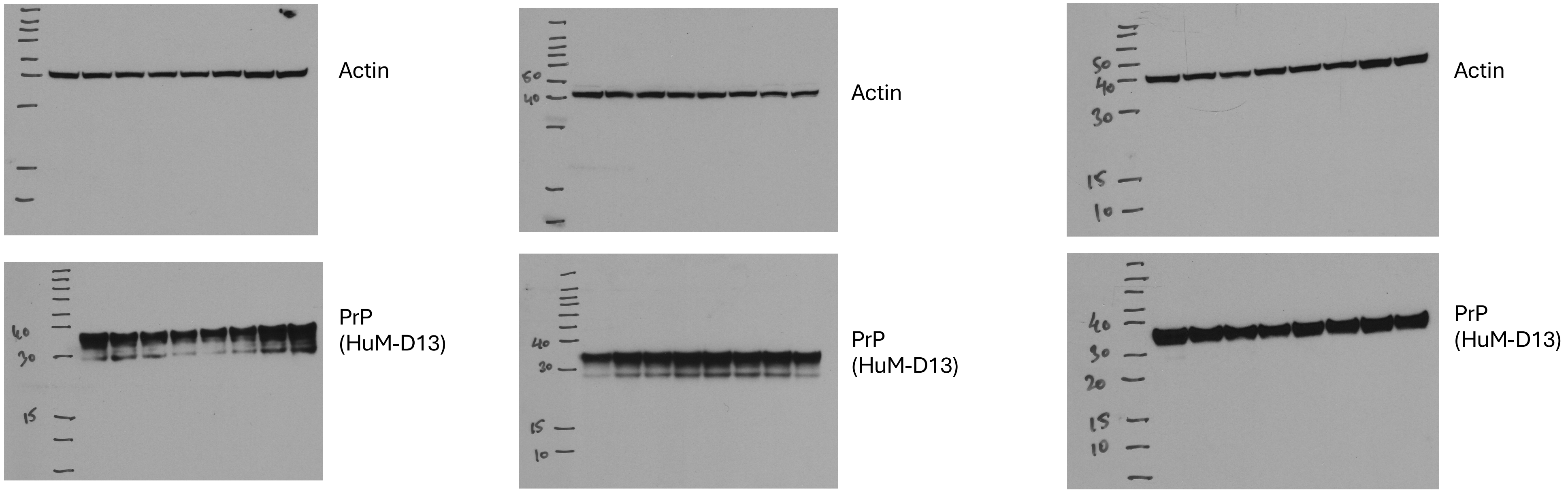

B

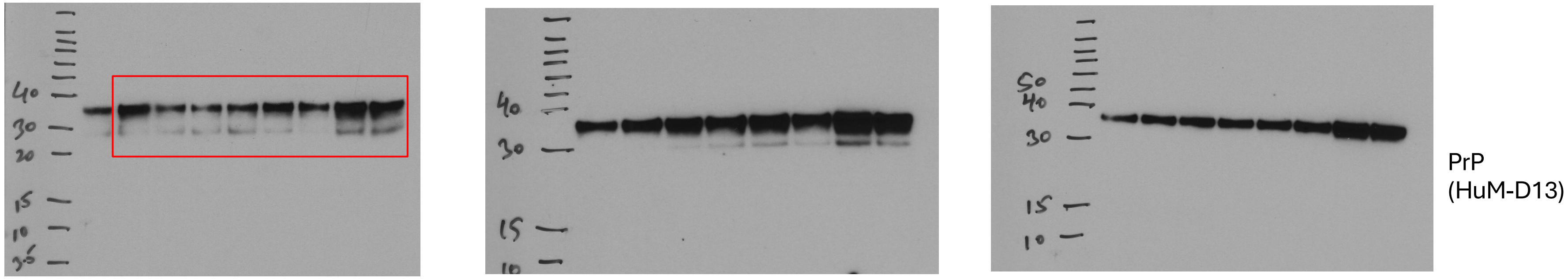

Full unedited blots for Supplementary Figure 7

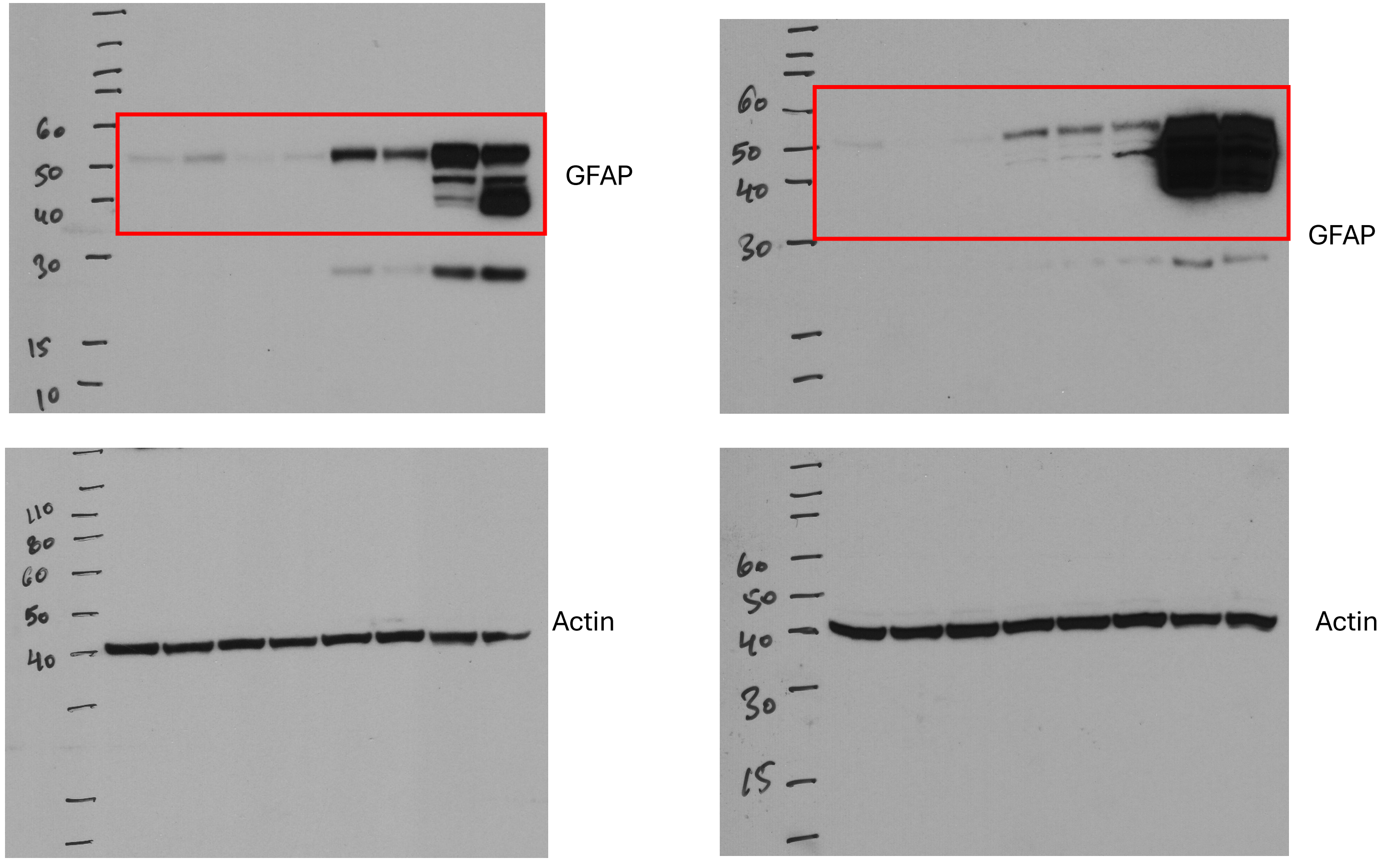

Full unedited blots for Supplementary Figure 8

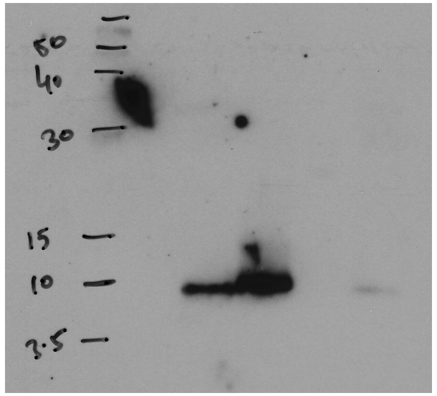

PrP
(HuM-D13)

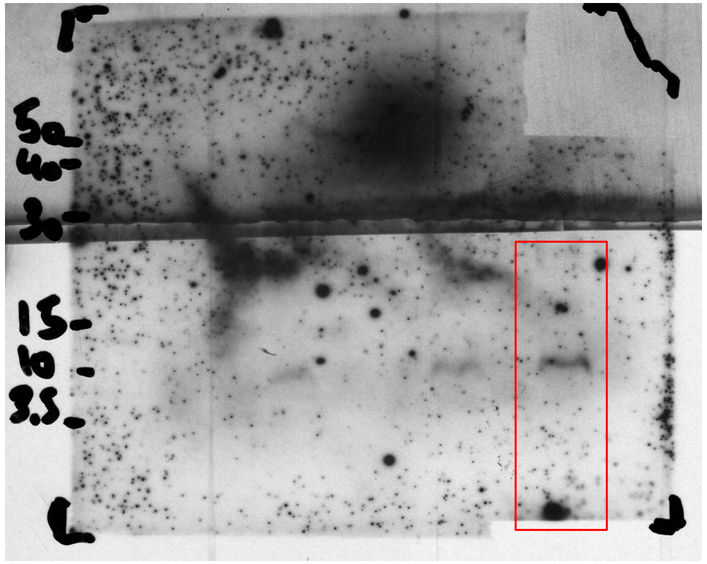

PrP
(HuM-P)

Full unedited blots for Supplementary Figure 9

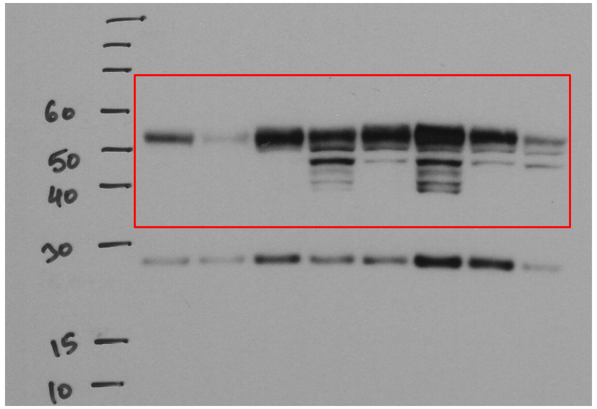

GFAP

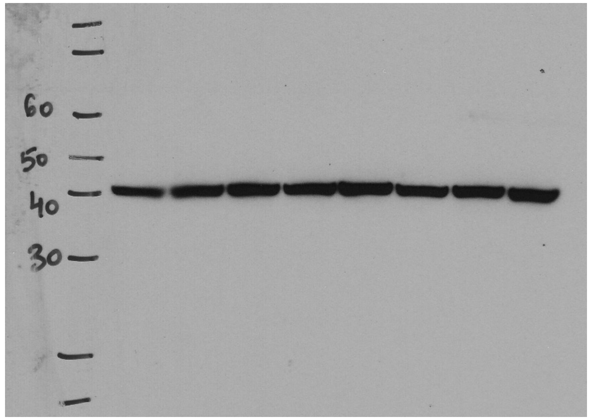

Actin
